# Spatial and Functional Heterogeneities Shape Collective Behavior of Tumor-Immune Networks

**DOI:** 10.1371/journal.pcbi.1004181

**Published:** 2015-04-23

**Authors:** Daniel K. Wells, Yishan Chuang, Louis M. Knapp, Dirk Brockmann, William L. Kath, Joshua N. Leonard

**Affiliations:** 1 Department of Engineering Sciences and Applied Mathematics, Northwestern University, Evanston, Illinois, United States of America; 2 Northwestern University Physical Sciences-Oncology Center, Evanston, Illinois, United States of America; 3 Department of Chemical and Biological Engineering, Northwestern University, Evanston, Illinois, United States of America; 4 Robert H. Lurie Comprehensive Cancer Center, Northwestern University, Evanston, Illinois, United States of America; 5 Northwestern Institute on Complex Science, Northwestern University, Evanston, Illinois, United States of America; 6 Institute for Theoretical Biology, Humboldt University Berlin, Berlin, Germany; 7 Chemistry of Life Processes Institute, Northwestern University, Evanston, Illinois, United States of America; National Institutes of Health, United States of America

## Abstract

Tumor growth involves a dynamic interplay between cancer cells and host cells, which collectively form a tumor microenvironmental network that either suppresses or promotes tumor growth under different conditions. The transition from tumor suppression to tumor promotion is mediated by a tumor-induced shift in the local immune state, and despite the clinical challenge this shift poses, little is known about how such dysfunctional immune states are initiated. Clinical and experimental observations have indicated that differences in both the composition and spatial distribution of different cell types and/or signaling molecules within the tumor microenvironment can strongly impact tumor pathogenesis and ultimately patient prognosis. How such “functional” and “spatial” heterogeneities confer such effects, however, is not known. To investigate these phenomena at a level currently inaccessible by direct observation, we developed a computational model of a nascent metastatic tumor capturing salient features of known tumor-immune interactions that faithfully recapitulates key features of existing experimental observations. Surprisingly, over a wide range of model formulations, we observed that heterogeneity in both spatial organization and cell phenotype drove the emergence of immunosuppressive network states. We determined that this observation is general and robust to parameter choice by developing a systems-level sensitivity analysis technique, and we extended this analysis to generate other parameter-independent, experimentally testable hypotheses. Lastly, we leveraged this model as an in silico test bed to evaluate potential strategies for engineering cell-based therapies to overcome tumor associated immune dysfunction and thereby identified modes of immune modulation predicted to be most effective. Collectively, this work establishes a new integrated framework for investigating and modulating tumor-immune networks and provides insights into how such interactions may shape early stages of tumor formation.

## Author Summary

Over the course of tumor growth, cancer cells interact with normal cells via processes that are difficult to understand by experiment alone. This challenge is particularly pronounced at early stages of tumor formation, when experimental observation is most limited. Elucidating such interactions could inform both understanding of cancer and clinical practice. To address this need we developed a computational model capturing the current understanding of how individual metastatic tumor cells and immune cells sense and contribute to the tumor environment, which in turn enabled us to investigate the complex, collective behavior of these systems. Surprisingly, we discovered that tumor escape from immune control was enhanced by the existence of small differences (or heterogeneities) in the responses of individual immune cells to their environment, as well as by heterogeneities in the way that cells and the molecules they secrete are arranged in space. These conclusions held true over a range of model formulations, suggesting that this is a general feature of these tumor-immune networks. Finally, we used this model as a test bed to evaluate potential strategies for enhancing immunological control of early tumors, ultimately predicting that specifically modulating tumor-associated immune dysfunction may be more effective than simply enhanced tumor killing.

## Introduction

Growth and persistence of a tumor is influenced not only by the intrinsic proliferative capacity of the cancer cells, but also by the complex ecosystem of cells, signaling molecules and vasculature surrounding the tumor, which collectively comprise the tumor microenvironment (TME) [1,2] An important feature of the TME is the important role played by non-tumor cells, including both immune cells and stromal cells, in promoting tumor proliferation by contributing to immune evasion, induction of angiogenesis, and other hallmarks of cancer [3,4]. The collective behavior of this coupled multicellular network plays a critical role in patient prognosis and the development of drug resistance, and the TME could ultimately serve as an attractive new target for treatment development [5–7]. Although many cell types and signaling molecules participating in these networks have been identified, our understanding of how these dysfunctional, tumor-facilitating network states are established, maintained, and potentially may be disrupted still remains limited.

In particular, while the composition of the late-stage TME has been extensively characterized in both animal models and patient samples, substantially less is known about how new TME states are established at sites of neoplastic growth or metastasis [8–11]. This lack of understanding is due in part to the difficulty in experimentally observing these processes. In both patients and model animals, for example, new metastatic sites are not typically identifiable until substantial growth has occurred, at which point the TME exhibits markers of a pro-tumoral state, including enhanced vascularization and the presence of immunosuppressive cells such as myeloid-derived suppressor cells, tumor associated macrophages and regulatory T cells. Conversely, the immune surveillance theory of cancer predicts that some neoplasms and metastases are controlled by the immune response [12], and moreover, it is widely hypothesized that the initial response to cancer is inflammatory and immunostimulatory [13,14]. Thus, although the early TME may switch from a tumor-controlling state to a tumor-promoting state [15–17], whether such a network state change occurs and the mechanisms that would drive such a shift remain unclear.

One physiological trait that may play a key role in such a shift is the capacity of immune cells to adopt different functional states in different environments, which is perhaps best illustrated by a cell type that strongly affects the early TME—the macrophage. As ubiquitous cells of the innate immune system, macrophages are present at the earliest stages of tumor establishment, are found in great numbers at tumor sites (along with the related myeloid-derived suppressor cells), and have the capacity to functionally “polarize” to phenotypes that alternatively suppress or promote tumor growth [18–20]. While macrophage polarization states likely comprise a continuum of phenotypes, these states may be broadly classified as either M1 –“classically activated” states characterized by inflammatory, tumor-suppressive phenotypes—or M2 –“alternatively activated” states characterized by anti-inflammatory phenotypes, which promote tumor survival and growth [18,21–24]. Thus, interactions between functionally heterogeneous immune cells, the tumor, and the TME may collectively drive dynamics of the early TME immune network.

A final feature of the TME that is likely to influence the evolution of network states is the important role of spatial organization and heterogeneity. Microenvironmental heterogeneities cause cells to experience different milieus within different areas of the tumor, and such heterogeneities may play a role in driving cancer pathogenesis and response to cancer therapy [25]. For example, hypoxic pockets in tumors can promote the survival of cancer cells during chemotherapy [26], and local gradients of key chemokines regulate chemotaxis of tumor cells and other cells in the TME to drive processes including tissue reorganization and invasion [27]. Together, local variations in microenvironmental chemokines, angiogenic factors, and tumor-invading cell types comprise a signature termed the “immune contexture,” which correlates with patient prognosis [28–30]. However, the causal underpinnings of such spatial heterogeneities is unclear, as is the degree to which they impact functional heterogeneities in immune cell states.

Given the aforementioned challenges associated with investigating TME dynamics experimentally, especially at the early stages of cancer initiation and progression, a complementary strategy is the use of computational modeling. A model that captures the proposed salient features of a system serves as a test bed for experimentation *in silico*, which can build understanding and guide formulation of experimentally-testable hypotheses [31]. Given the importance of spatial features and varying cell states and types in the TME, a suitable framework is a hybrid discrete-continuous (HDC) agent based model. In these models, individual cells in the system are represented as “agents” on a lattice (typically 2-dimensional), each of which is capable of moving, making decisions, and interacting with other cells via diffusible signals [32,33]. Such models have been extensively employed to facilitate qualitative understanding of various phenomena in cancer. For example, a two dimensional model of tumor invasion demonstrated that a harsh and/or heterogeneous tumor microenvironment preferentially selects for aggressive cancer phenotypes [34]. Another HDC model predicted that in contradiction to the prevailing conceptual model, production of transforming growth factor-β by tumor and stromal cells would not increase the likelihood of tumor survival, a prediction that was later experimentally verified [35]. An HDC model of cancer stem cells was also used to identify phenotypic traits that are most important for tumor initiation and highlighted the impact of microenvironmental effects on individual cellular behaviors [36]. Such approaches have also been used to develop patient-specific treatment strategies [37,38]. A key feature of this modeling approach is that cellular behaviors, such as chemotaxis or cell division, are typically encoded using “rules” or input/output relationships that effectively distill the intracellular molecular mechanisms that lead to such behaviors into their simplest form. To date, such modeling has not been applied to investigating the dynamics of tumor-immune interactions within the TME, and this approach is ideally suited to investigating the coupled roles of the spatial organization and functional heterogeneity described above.

In this study, we used a computational approach to elucidate general principles by which heterogeneities in the spatial structure of the TME and in immune cell phenotype may drive the dynamic evolution of the collective immune response to a nascent metastatic tumor. In particular, we investigated the transition from an initially immunostimulatory state to an immunosuppressive, tumor-promoting state during the earliest times of tumor progression (within 5 days of implantation). Towards this goal, we developed an HDC model that incorporates both salient spatial features of the TME and biologically realistic models of macrophage functional polarization, which capture our developing understanding of the process by which these key players in early TME immunity make decisions. A central innovation of this investigation was the development and application of system-wide multiparametric sensitivity analyses of our HDC models, effectively providing parameter-independent understanding of tumor-immune interactions in the TME and establishing useful metrics for systems-level analysis of such models. These investigations indicated a central role for heterogeneities in both spatial organization and cell phenotype in driving the emergence of immunosuppressive network states. Finally, we harnessed this model as an *in silico* test bed to evaluate and compare potential strategies for engineering novel cell-based therapies that may disrupt immunosuppressive networks and drive tumor clearance [39–41]. Ultimately, this approach generates clinically relevant and experimentally testable hypotheses and could establish a foundation for integrating additional immunological details to elucidate processes of greater complexity and address later stages of TME development.

## Models

Overall, we sought to investigate the questions posed above using the simplest, minimally parameterized model that adequately describes core features of tumor-immune dynamics at a novel tumor site. Core features and assumptions of our model were as follows: we assumed that early immune dynamics following implantation of metastatic tumor cells into a pre-existing tissue are largely governed and/or represented by macrophages (including both tissue-resident macrophages and those that enter via the vasculature, for example by differentiating from circulating monocytes) [13,14,19,42], and thus our model of early TME development omits other cell types. This macrophage-centric view is also supported by the high prognostic value of tumor-associated macrophage phenotypes for predicting cancer progression [43]. We incorporated mechanisms for macrophage chemotaxis, macrophage functional polarization to M1 or M2 states, macrophage-mediated tumor killing, and tumor necrosis-mediated activation of macrophages via release of soluble factors, along with mechanisms for oxygen uptake by cells, oxygen delivery via vasculature, angiogenesis mediated by release of pro-angiogenic factors, and hypoxia-mediated cancer cell death. In general, our governing principle was to reduce biological complexity by describing modeled processes with “representative” soluble factors and cell states, in order to capture general behaviors of the system rather than pathway-specific details. Complete details and rationale for all modeling choices are presented in S1 Text.

## Results

**Fig 1:**
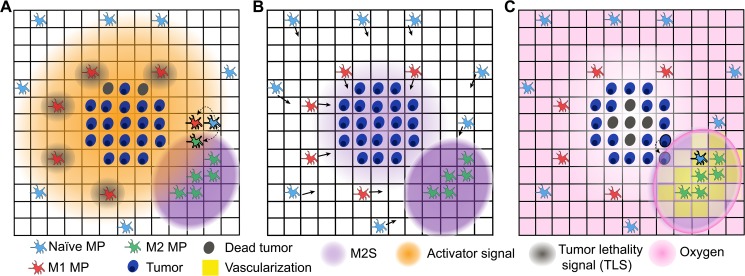
Core features of the early tumor-immune network model. This schematic illustrates key processes captured in the HDC agent-based model. (A) *Macrophage polarization and tumor killing*. Naïve macrophages (MP) polarize to either an M1 state, in the presence of high levels of Activator signal, or an M2 state, with concomitant exposure to M2 signal (M2S). M1 cells secrete tumor lethality signal (TLS), high levels of which kills tumor cells. (B) *Macrophage chemotaxis*. All macrophages chemotax along gradients of M2S, which is secreted by the tumor and M2 cells. (C) *Vascularization*, *tumor proliferation*, *and the effects of oxygen*. Oxygen and naïve MP enter at sites of vascularization, which increases as a function of local levels of M2S. All cells die in anoxic conditions, and dead cells are retained (e.g., forming a necrotic tumor core, as depicted). Individual tumor cells divide at a fixed rate, expanding to “invade” neighboring lattice sites.

**Fig 2:**
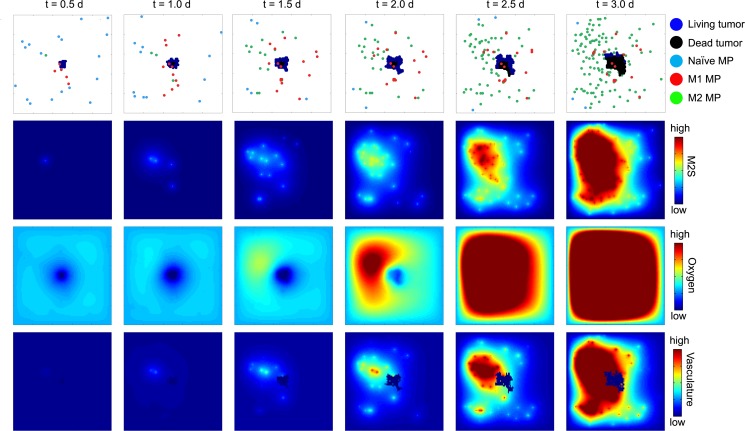
Qualitative recapitulation of tumor physiology. Qualitative behavior of the model is illustrated through snapshots in time from a single run of the HDC model using base parameter values. At each time point post-tumor initiation (in days), spatial distributions of cells (by type), M2S, oxygen, and vasculature are depicted. In this particular run, shortly after the final time point shown, all tumor cells were dead. Numerical ranges spanned by colored scale bars are: M2S (0–4 [pg/LS] x 10^–6^), oxygen (0–1 [pg/LS]), vasculature (0–40 au).

Fig. 1 provides a conceptual overview of the processes governing the five classes of cellular agent, four diffusible signals and vasculature in our model. Interactions between different agents via the diffusible signals and vasculature include polarization of naïve macrophages to either M1 or M2 phenotypes and tumor cell killing by M1 cells (Fig. 1A), macrophage chemotaxis along a chemokine gradient (Fig. 1B), and the effects of oxygen and vascularization on both tumor growth and death, as well as on macrophage recruitment (Fig. 1C). Each such interaction is depicted explicitly in S1 Fig, and S2 Fig summarizes the algorithmic approach by which our simulations were implemented. We first verified that such a framework encapsulates sufficient biological detail to recapitulate known qualitative features of tumor biology. For example, Fig. 2 depicts a single simulation of the model run under base parameter values, and illustrates several key features. Initial oxygen uptake resulted in a hypoxic environment and the formation of a central necrotic core (t < 2.0 days), which is an established feature of solid tumors [44]. The resulting limited oxygen availability led to asymmetric tumor growth, which is also a general feature of growing tumors [34,45]. As M2-polarized macrophages arose and promoted vascularization, the resulting oxygenation limited growth of the necrotic core. Extensive invasion of the tumor by macrophages was observed at later time points, which is consistent with experimental observations [30]. Notably, this particular simulation run resulted in tumor death due to early M1-mediated responses, despite the prevalence of M2 cells at later time points, suggesting that the model captured elements of the competition between immune surveillance and immune dysfunction [12]. Overall, this framework appeared to be biologically realistic and was used to investigate the motivating questions posed above.

### Functional and spatial predictors of tumor clearance

We first investigated how functional and spatial features of the TME contribute to the dynamic evolution of tumor-associated immunity and tumor clearance. The HDC framework introduces spatial heterogeneity (i.e., spatial variations in concentration) for each of the four diffusible signals. These concentration fields evolve over time and impact the microenvironment to which an incoming naïve macrophage is exposed (Fig. 2). To investigate one additional potential source of functional heterogeneity, we hypothesized that macrophages may polarize towards M1 or M2 states in a *stochastic* manner, such that the *probability* of adopting an M2 state is increased in proportion to environmental pro-M2 signal, and thus this decision is not a deterministic function of pro-M2 signal concentration. In our model, the pro-M2 signal is denoted “M2S”, which is a “lumped” soluble factor that captures the fact that M2 cells and tumor cells secrete M2-inducing cytokines, pro-angiogenic factors, and chemoattractant signals (see S1 Text). This hypothesis of stochastic polarization is inspired by observations that macrophage gene expression in response to certain stimuli exhibits stochasticity when quantified at the single cell level [46,47]. Thus, we first characterized the dynamic evolution of the TME network under conditions that modulate the manner in which macrophages “decide” to adopt one functional state or another.

**Fig 3:**
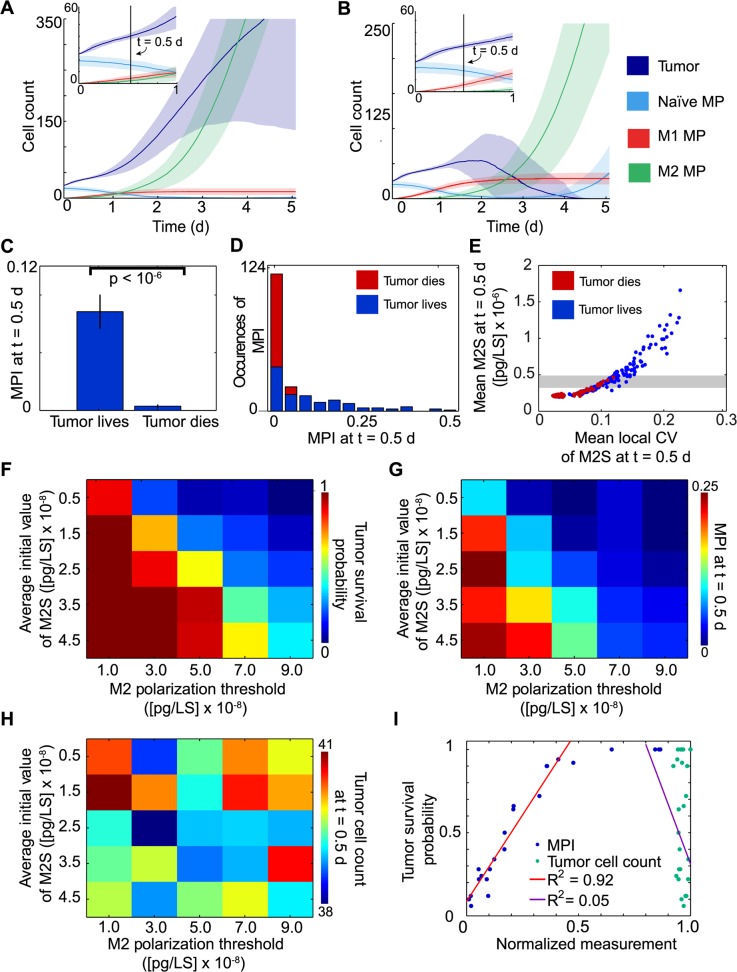
Functional and spatial predictors of tumor clearance. The model was run 200 times at base parameter values, and runs were grouped based upon simulation outcome (tumor survival or tumor death). (A,B) Time evolution of cell counts (by type) for runs in which the tumor survived (A) or died (B). *Insets*: Expanded views of early time points (same axes). (C) Macrophage polarization index (MPI) at 0.5 d post tumor-initiation, classified by simulation outcome. Error bars indicate standard error. (D) Distributions of MPI at t = 0.5 d observed across multiple simulations, classified by outcome. (E) Spatial metrics of the TME at t = 0.5 d: domain-wide maximum M2S value and mean local coefficient of variation (CV) of M2S, classified by outcome. Local CV of M2S was calculated within each 10 x 10 lattice site (LS) array, and mean of all 100 such arrays across the domain is shown. (F) Tumor survival probability evaluated across variations in average initial M2S level (*p11*) and M2 polarization threshold (*p13*). (G) MPI at t = 0.5 d evaluated across the same parameter variations used in F. (H) Tumor cell counts at t = 0.5 d evaluated across the same parameter values used in F. (I) Normalized MPI (blue) and normalized tumor cell count (green), both at t = 0.5 d, calculated across the same parameter values used in F and plotted against tumor survival probability. Linear regression was performed on data points for which tumor survival probability was less than 1.

Each run of an HDC simulation is stochastic, meaning that many aspects of the model are pseudo-random with respect to both the initiation and time evolution of each simulation run. In particular, the initial spatial distribution of macrophages was uniformly random throughout the environment, which initially represents tissue imbued with a complement of tissue-resident macrophages and vasculature providing blood to the tissue. New macrophages were introduced to sites within the tissue lattice stochastically, in proportion to the level of vascularization at each site, in order to capture the fact that increased vascularization leads to increased infiltration of new macrophages from progenitors in the blood. The initial concentration of M2S at each lattice site was also randomly selected from a pre-specified normal distribution, in order to capture the potential for spatial variation (spatial heterogeneity) in the initial M2S concentration within tissue. Lastly, as discussed above, macrophage functional polarization to one state or another was also stochastic, with the likelihood of polarizing to the M2 state determined by the local concentration of M2S. Further details are provided in S1 Text. Given the inclusion of these various elements of chance, assessing model behavior under a given set of parameters necessitated generating a series of simulations run under identical parameter values, and then quantifying simulation outcome using a relevant metric. For example, simulation outcomes may be broadly categorized into runs resulting in, alternatively, tumor survival (i.e., escape from immune control) or tumor death—these two outcomes are illustrated in S1 Movie and S3 Movie, respectively (note that each movie is published in two file formats, and herein we refer only to the first instance of each movie). To characterize model behavior under base parameter values, the simulation was run 200 times, and outcomes were classified as either tumor survival (greater than 0 tumor cells at t = 5 d) or tumor death (0 tumor cells at t = 5 d). Notably, both outcomes were observed under our base parameter values, and the averaged time courses of tumor volume and macrophage cell count for simulation runs subdivided into these two outcomes are shown in Fig. 3 panels A and B, respectively. Interestingly, the average time required to establish a pro-tumor microenvironment (large population of M2 cells) differed substantially between cases resulting in tumor survival (Fig. 3A) vs. tumor death (Fig. 3B), with pro-tumor TME formation occurring almost immediately in the cases of tumor survival. Cases of tumor survival also exhibited a substantially larger M2 response, while a large M1 response appeared to precede cases of tumor death (see green and red curves in Fig. 3A,B). Nearly identical behavior was observed when we introduced a stochastic component into the rule governing macrophage chemotaxis [34] (S3 Fig), and for this reason we used a deterministic model of chemotaxis in subsequent simulations and analyses. In all cases, there was eventually a switch from a tumor suppressing, M1-dominated state to a tumor-promoting M2-dominated state. Notably, this switch was mediated only through differential polarization of newly recruited macrophages, rather than conversion of macrophages from one state to another, as has been reported to occur under some circumstances [48,49] but which was not permitted in our model. In our model, tumor-mediated manipulation of macrophage “plasticity” was not necessary for the establishment of an immunosuppressive microenvironment.

To further analyze the observed differences in immune response between cases resulting in tumor death vs. survival, we defined a statistical metric for assessing the relative prevalence of M2 cells within the overall macrophage population. We termed this metric the “macrophage polarization index” (MPI), which is evaluated at a particular point in time as follows:

$$\text{MPI}\equiv \frac{\text{M2 count}}{\text{1 + M1 + naïve macrophage count}}$$

As a ratio of M2 cells to all other cells types, the MPI reflects the overall bias of the population towards the M2 phenotype, since it decreases with prevalence of either M1 cells or naïve macrophages. The mean MPI at t = 0.5 d was calculated for simulations resulting in tumor survival vs. tumor death (Fig. 3C). Even though the tumor cell count in each of these cases was indistinguishable at this time point (see insets of Fig. 3A,B), the MPI was significantly different between the two cases (p < 10^–6^). In general, the MPI at this early time point was highly predictive of the ultimate outcome of the simulation (Fig. 3D).

To investigate whether spatial features of the TME were also predictive of tumor survival at the time at which outcome appeared to become deterministic (i.e., t = 0.5 d), we used the base parameter values (those used for Fig. 3A-D) and quantified the degree to which various metrics of spatial features correlated with tumor survival. We first evaluated two spatial metrics related to M2S at t = 0.5 d: the “local” coefficient of variation (CV) of M2S and the mean amount of M2S present throughout the domain (Fig. 3E). Local CV was defined as the CV of M2S within a 10 x 10 array of adjacent lattice sites (LS), and local CV values were then averaged over all 100 such arrays within the domain. Beyond a certain threshold in either metric, tumor eradication was not observed. Notably, within an envelope indicated by a gray bar in Fig. 3E and for any value of mean M2S, tumor survival was more likely for larger values of mean local CV of M2S. A similar effect was observed when comparing domain-wide maximum M2S with mean local CV of M2S (S4 Fig). Together, these results may suggest that local pockets of elevated M2S may promote tumor survival at early stages of tumor formation while the system gradually shifts to an M2-dominated state overall.

We next evaluated whether the early immune response, as measured by the MPI, remains highly predictive of tumor survival over a range of model parameterizations. We systematically varied the two (out of eighteen) model parameters expected to most bias the propensity of the system to adopt an M2-dominated state—average initial M2 signal (*p11*) and the M2 polarization threshold (*p13)*. This focused multiparametric sensitivity analysis revealed that MPI at t = 0.5 correlated with tumor survival probability across the range of parameters evaluated (Fig. 3F,G), whereas neither parameter correlated with tumor size at that time (Fig. 3H). Quantification of these trends confirmed high correlation of tumor survival and MPI (t = 0.5 d) (R^2^ = 0.92) vs. low correlation between tumor survival and tumor size (t = 0.5 d) (R^2^ = 0.05) over various model parameterizations (Fig. 3I). Thus, a general feature of our simulations was that tumor survival or death is a nearly deterministic consequence of the early immune response long before growth of the tumor is impacted.

### Systematic characterization of TME network robustness and the role of heterogeneities

**Fig 4:**
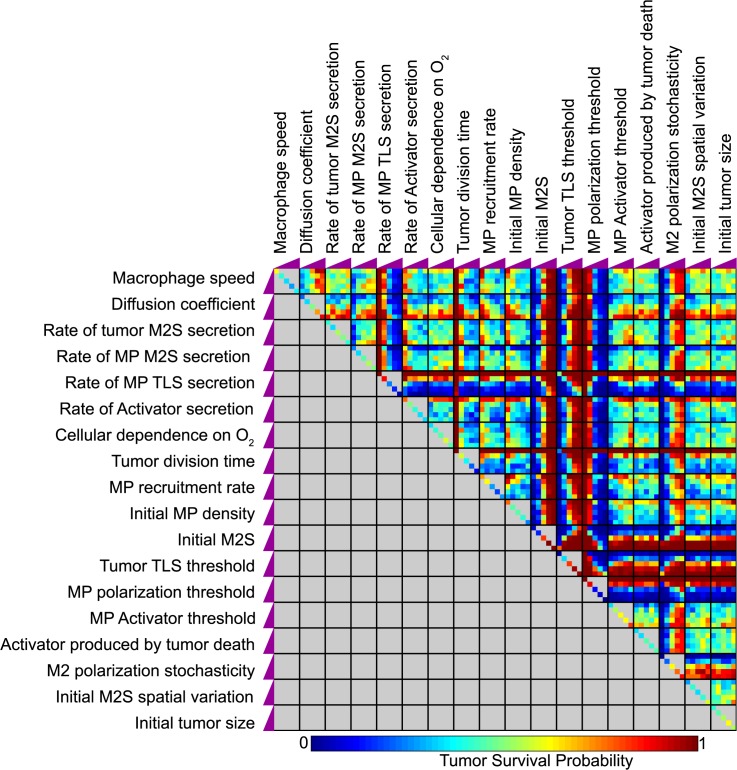
Systematic multi-parameter sensitivity analysis (MPSA). Parameter sensitivity was evaluated by running simulations using 5 values of each parameter, spanning a single order of magnitude around the base value (see text), and this was performed for each combination of values for each parameter pair. 50 simulation runs were performed for each set of parameter values, and the tally of simulation outcomes is indicated by the color. The change in each parameter magnitude across its range is indicated by its corresponding purple ramps on the boundaries of the plot.

To better understand the processes driving TME evolution and tumor survival in a manner that is less sensitive to our particular model formulation, we required a method for dissecting the influences of both individual parameters and combinations of parameters. For models based upon differential equations, this task can be accomplished by multiparametric sensitivity analyses (MPSA), which can identify combinatorial influences of multiple parameters and elucidate systemic features of such a model, such as the boundaries of distinct regimes of model behavior [50]. To analyze our model in this way we developed a highly parallelizable two-dimensional MPSA approach, which to the best of our knowledge is the first time such a systematic analysis of parameter sensitivities has been undertaken for an HDC model. For each individual parameter (18 cases) and each unique pair of parameters (171 cases), simulations were run using parameter values distributed as follows:

$$\left[\text{0.25, 0.75, 1.25, 1.75, 2.25}\right]*p_{i}$$

where *p*_*i*_ is base value of parameter *i*. This linear distribution of values near the base case was selected to generate a robust evaluation of sensitivity for parameter values that are most physically reasonable (base case values were estimated based upon physical measurements and reasoning; see S1 Table). To also evaluate parametric sensitivity in the context of greater fold-deviations from the base case, we performed a second MPSA using a broader, more sparsely sampled range of parameter values that covered two orders of magnitude (S5 Fig). With a few exceptions (detailed in the caption of S5 Fig), the sensitivity of simulation outcomes to the parameters was found to be less informative over this larger parameter set, and for this reason we focused our analysis on the initial parameter set (above). Each scenario was simulated 50 times under identical parameter values, and each simulation run was classified by outcome (i.e., tumor survival or tumor death) (Fig. 4). An immediate high-level observation is that chance played a important role in tumor survival across a wide range of parameter values and combinations thereof—out of the 3915 unique parameter combinations simulated, only 439 (11.2%) resulted in either 100% tumor survival or death.

**Fig 5:**
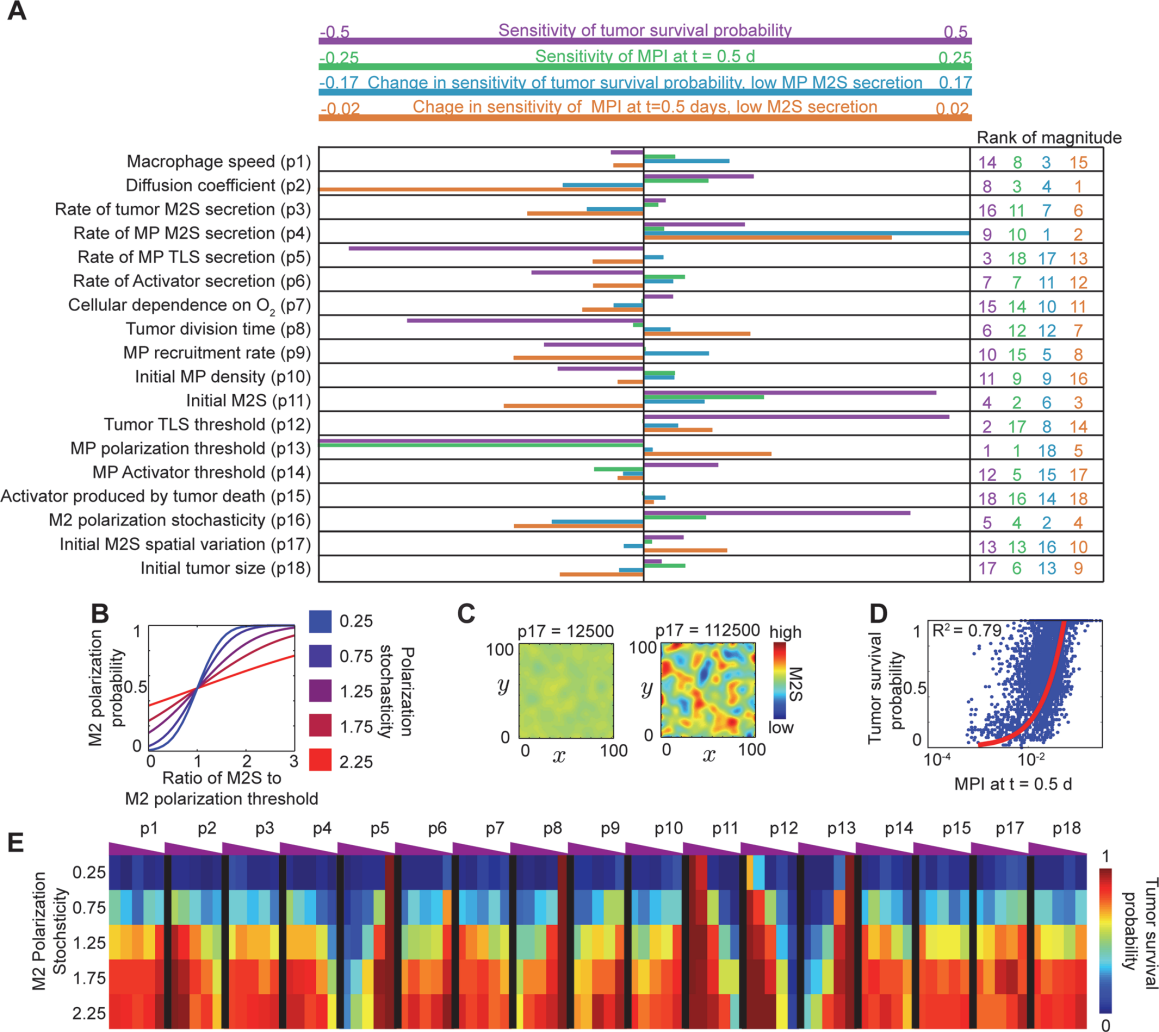
Systematic evaluation of parameter contributions to model behaviors. (A) Using the data from Fig. 4, sensitivities of four different metrics of model behavior were calculated over changes in each of the 18 model parameters. The range of each metric was different (indicated in key at top of panel). For each metric, normalized sensitivities were ranked by parameter (right column). (B) Illustration of M2 polarization probability “dose-response” curves (normalized) for different values of polarization stochasticity. (C) Illustrative spatial distributions of M2S for low and high values of the M2S heterogeneity parameter, *p*17, depicted at 0.1 d after tumor initiation, after which point initial diffusion had occurred but cellular contributions to the distribution were negligible. (D) Global correlation of tumor survival probability with MPI at t = 0.5 d across all parameter combinations evaluated in Fig. 4. Red lines depict linear regressions, with coefficients shown. (E) Pairwise contributions of polarization stochasticity and all other parameter values to tumor survival probability.

To systematically quantify the degree to which each parameter contributed to model behaviors, such as tumor survival, under all one- and two-parameter variations evaluated in our MPSA (Fig. 4), we built a non-linear (quadratic) regression model and applied it to these data (Fig. 5A). Such a regression model is among the simplest ways to evaluate parameter influence—a quadratic equation is fit to (regressed against) the MPSA data to generate the best possible prediction of model outcome (e.g., probability of tumor survival) as a function of parameter value. The partial derivative of this equation with respect to this parameter is evaluated and defined as the sensitivity of this model output to the parameter under consideration (this derivative is typically evaluated at the base parameter value). The resulting sensitivities of tumor survival probability to each parameter are shown as purple bars in Fig. 5A. One immediately surprising result of this analysis was that increases to either functional heterogeneity (macrophage polarization stochasticity, *p16*, shown in Fig. 5B) or spatial heterogeneity (variation in the spatial distribution of initial M2S concentration, *p17*, shown in Fig. 5C) led to increases in tumor survival. Together, these results suggest that, in general, increased heterogeneity (both spatial and functional) enhanced tumor survival.

To investigate the mechanism through which these types of heterogeneity increased the chance of tumor survival, we next examined the sensitivity of an additional simulation outcome metric, the MPI at t = 0.5 d (which was shown above to predict tumor survival outcome), to changes in each parameter value. As was previously observed (Fig. 3H), the MPI at this time correlated strongly with tumor survival probability across all variations of the parameters (R^2^ = 0.79, shown in Fig. 5C), indicating that this metric was a robust predictor of tumor survival in our model. As shown in Fig. 5A (green bars), the MPI sensitivity to changes in either M2 polarization stochasticity or initial M2S spatial variation was positive, indicating that one possible mechanism through which functional and spatial heterogeneity contributed to tumor survival was by increasing the MPI at an early time point. This early increase in MPI effectively lessened the probability of achieving a uniform immunostimulatory state and accelerated the emergence of immunosuppression. This prediction is illustrated in S5 Movie and S7 Movie, which depict simulations run under conditions of high spatial (S5 Movie) and functional (S7 Movie) heterogeneity, and which exhibit accelerated onset of system-wide immunosuppression throughout the TME.

We hypothesized that if spatial and functional heterogeneity led to locally elevated concentrations of M2S, which in turn induced a local population of macrophages to reinforce and disseminate their M2 state via positive feedback mediated by M2S, then reducing the rate at which M2S is secreted by M2 cells could mitigate the pro-tumor effects of increased heterogeneity. To evaluate this hypothesis, we recalculated the sensitivities of tumor survival probability and the MPI at t = 0.5 days for all parameters, while setting the rate at which M2 cells secrete M2S to a low value (*p4* = 0.25) as the new base case. The *changes* in sensitivities (relative to those calculated for the original base case, where *p4* = 1.0) are shown in blue for tumor survival probability and orange for MPI at t = 0.5 days in Fig. 5A. Notably, in this case of reduced M2S secretion, the sensitivity of tumor survival to both functional heterogeneity (*p16*) and spatial heterogeneity (*p17*) decreased, which is consistent with our hypothesis and suggests that increased M2S secretion can interact synergistically with both spatial and functional heterogeneity to increase tumor survival. Interestingly, however, the sensitivity of the MPI to increases in spatial and functional heterogeneity did not follow this pattern; in particular, while the MPI sensitivity to *p16* decreased (as expected), the MPI sensitivity to *p17* increased. These results suggest that MPI may serve as a less robust predictor of tumor survival in the context of reduced M2S feedback (S6 Fig).

The synergistic relationship between functional heterogeneity and M2S secretion by M2 cells is further demonstrated in Fig. 5E, which shows how polarization stochasticity (*p16*) interacts with the other 17 parameters in the model to influence tumor survival probability. High polarization stochasticity generally produced high tumor survival probabilities, and this effect was only prevented in a few cases, perhaps most notably, when M2S secretion by M2 cells (*p4)* was decreased. Thus, our model predicts that one possible strategy to reduce the pro-tumor effects of heterogeneity may be to inhibit feedback mechanisms that reinforce the immunosuppressive state. This prediction is illustrated in S5 Movie, which depicts a simulation of the model run under decreased M2 feedback and increased functional heterogeneity, in which the lack of M2 feedback gives the system more time to achieve a uniformly immunostimulatory state that ultimately eliminates the tumor.

### Evaluation of potential engineered cell-based therapy strategies

Building on insights gleaned from model analysis, we next used this framework as an *in silico* test bed for evaluating potential therapeutic strategies for promoting immunological control of cancer in our early TME model. Here we focused on strategies comprising engineered cell-based therapies, which provide the unique capacity to interact with host physiology in a programmable fashion and have emerged as powerful, viable approaches for treating gastrointestinal diseases, obesity, and most notably cancer [51–53]. In order to minimize introduction of new modeling artifacts, we also focused our analysis on strategies in which macrophages are genetically engineered to act as “Trojan horses”—cells that retain and leverage intrinsic macrophage properties but are modified to interact with the TME in a novel, programmed fashion. Macrophages are indeed attractive candidates for cell-based therapy, given their native ability to localize to tumors and transient lifetime post-administration, which could reduce the consequences of immunological rejection and lower risks of genetic modification-mediated malignant transformation [54–56]. Finally, we focused our analysis on strategies that could feasibly be implemented by genetically engineering macrophages *ex vivo* (e.g., using autologous monocytes collected by apheresis), and then reintroducing engineered cells to a patient, with the expectation that engineered cells would likely comprise a minority of the macrophages present in the TME.

**Fig 6:**
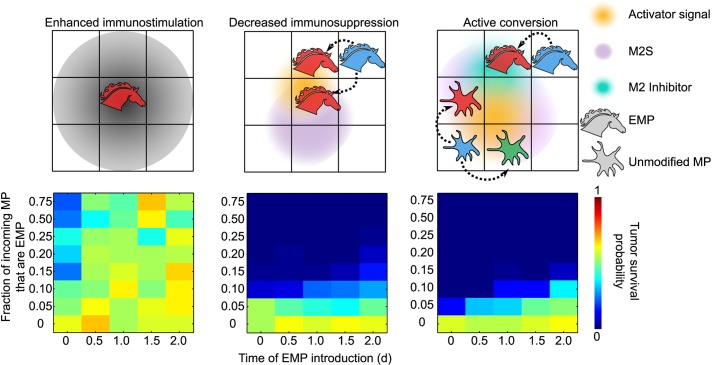
Evaluation of potential engineered cell-based therapy strategies. Three potential strategies to treat cancer with engineered macrophages (EMP) were evaluated for efficacy in promoting tumor clearance in this model of the early TME. Enhanced immunostimulation (left column): when polarized to the M1 state, EMP released four times the base case level of TLS. Decreased immunosuppression (center column): EMP could not polarize to the M2 state. Active conversion (right column): EMP could not polarize to the M2 state and constitutively secreted a diffusible signal that blocked the M2 receptor on normal (unmodified) macrophages. For each strategy, tumor survival probability was calculated over simulations varying the time at which the first EMP were introduced and the fraction of incoming MP that were MP (vs. unmodified); 50 simulations were performed for each case.

Based upon our system-wide parameter sensitivity analysis (Fig. 5A), we proposed and evaluated three strategies for implementing Trojan horse-like engineered macrophages (EMP). In the first, EMP polarized to M1 and M2 states as did normal cells, but upon polarizing to the M1 state, EMP secreted the “tumor lethality signal” (TLS) at 4 times the normal rate. This enhanced immunostimulation strategy is illustrated in Fig. 6, left column. In the second strategy, EMP were prohibited from polarizing to the M2 state to implement decreased immunosuppression (Fig. 6, middle column). The third strategy was explicitly designed to drive conversion/reversion of the immunosuppressive TME to an immunostimulatory state, guided by our model analysis—EMP were prohibited from polarizing to an M2 state and also constitutively released a factor that blocked M2 signal-mediated polarization of naïve macrophages to the M2 state (Fig. 6, right column). In practice, such a blocking factor might represent a secreted monoclonal antibody or other protein antagonist that binds the receptor responsive to a pro-M2 cytokine (e.g., the IL-10 receptor).

The efficacy of each strategy was evaluated by examining cases varying both the time at which EMP were introduced (potentially allowing the establishment of an M2-dominated TME), and the fraction of incoming macrophages that were EMP vs. native cells. For each case, tumor survival probability across multiple simulation runs was quantified (Fig. 6, bottom row). Notably, EMP that simply released increased amounts of TLS were only efficacious if introduced immediately upon tumor implantation and in high frequencies compared to naïve macrophages (> 50%). In contrast, strategies relying on manipulating EMP and naïve macrophage polarization required substantially fewer EMP (<10% of incoming macrophages) and conferred efficacy when introduced at essentially any time. Thus, strategies focused on decreasing tumor-induced immunosuppression of macrophages were more efficacious than those focused on enhancing anti-tumor lethality of macrophages. Interestingly, there was little difference in tumor survival between the simpler strategy of blocking EMP polarization to the M2 state vs. the more complex strategy of engineering EMP to release a soluble factor blocking M2 polarization of naïve macrophages. Thus, in our model, it was sufficient to engineer macrophages to avert immunosuppression, and conversion of the overall TME was not required. Our analysis thus generated a number of experimentally testable therapeutic strategies and, as discussed below, may even concur with recent and early preclinical evaluation of therapeutic strategies that shift macrophage polarization [57].

## Discussion

The network of cells comprising a tumor and associated host cells is replete with spatial and functional heterogeneities that shape tumor-immune network dynamics and may ultimately impact tumor eradication or proliferation. Given the challenges associated with investigating such systems *in vivo*, particularly at early stages of tumor development, we developed a computational model serving as a both a conceptual tool and an *in silico* test bed for building understanding of such phenomena. Our analysis focused on capturing a reasonable topological description of the tumor-immune network at early stages of tumor establishment, such as may occur following neoplastic transformation or metastasis. This approach sacrificed some details in favor of enabling systematic analysis of the rich set of interaction dynamics within the TME. In particular, our model relied on a rule-based approach [34–36,58,59], in order to capture the core phenomena modeled, and thus it did not include explicit biochemical mechanisms describing processes such as signal transduction and gene regulation. To holistically evaluate model behavior, we developed a multi-parameter sensitivity analysis for characterizing our HDC model and generated systems-level, parameter-independent descriptions of the early tumor-immune network. Lastly, we harnessed this model as an *in silico* test bed for evaluating and comparing several intuitively attractive and clinically-implementable strategies for engineering cell-based therapies to facilitate cancer treatment. Taken together, this investigation illustrates a powerful approach by which computational tools may be harnessed to both build systems-level understanding from individual experimental observations, and then leverage this understanding to postulate experimentally testable hypotheses that can inform development of novel therapeutic strategies.

This investigation yielded several new insights into the roles that spatial and functional heterogeneities may play in the establishment and function of the TME. For example, under a wide variety of model parameterizations, the general structure of this system led to a prominent role for chance in determining whether a new tumor is ultimately eliminated or persists. Ultimately, the immunological state (or contexture) at the tumor site predicted tumor survival long before an impact on tumor growth was evident. Moreover, our systematic MPSA suggested that increased amounts of either functional and/or spatial heterogeneity increased tumor survival probability, possibly through a mechanism by which the occurrence of the first M2 macrophages was accelerated. This prediction complements reports that increased spatial heterogeneity in the TME vasculature can decrease the efficacy of chemotherapy [7,60]. This prediction also dovetails with observations that variability in the location, density and type of tumor-infiltrating immune cells has prognostic value [28]. Importantly, we observed that this result depended on the presence of a positive feedback mechanism for reinforcing the pro-tumor state (i.e., secretion of M2S by M2 cells). This observation indicates that in our model, intercellular communication and feedback were crucial for propagating the effects of heterogeneity throughout the TME. Based on our investigations, we would hypothesize that elevated spatial heterogeneity in the TME might correlate with severity of tumor-associated immune dysfunction or intransigence to therapeutic intervention, and it could be informative to investigate whether metrics defined on such a basis (e.g., degree of spatial clustering in tumor-infiltrating immune cells, which may be quantified histologically in tumor biopsy samples) might have prognostic value. Furthermore, we hypothesize that therapeutics that target pro-M2 feedback mechanisms within the TME may ameliorate the negative role of heterogeneity described here. This strategy may also dovetail with recent experimental evidence that therapeutic blockade of IL-10 enhances primary tumor responses to paclitaxel [61].

The synergistic interaction between heterogeneity in the TME and pro-tumor feedback could help to explain the increased occurrence of neoplastic and metastatic lesions in patients experiencing systemic immunosuppression, either through the action of established tumors at distal sites [24,62] or through the action of immunosuppressive drugs administered following organ transplantation [63]. If the establishment of a tumor-supporting local network state (vs. one that controls tumor growth) is supported through positive feedback, then any pro-tumor influence, even one that is stochastic, could tip the balance towards favoring an M2 phenotype that promotes tumor survival.

These conclusions were reinforced by our *in silico* evaluation of potential strategies for engineering macrophage cell-based therapies to overcome tumor-associated immune dysfunction. In particular, we observed that strategies that suppressed the emergence of an M2-dominated state were more effective than a strategy that simply enhanced the potency of macrophage-mediated tumor killing. More specifically, both strategies that suppressed the polarization of engineered macrophages to an M2 phenotype were effective at controlling tumor growth, even when these engineered cells comprised a minority of macrophages at the tumor site and polarization of native macrophages was not manipulated. Thus, we would predict that therapies that reduce the propensity of macrophages to polarize to M2 could be clinically promising. Indeed, this hypothesis is supported by a recent preclinical evaluation of a macrophage-targeted drug for treating glioblastoma multiforme (GBM) by antagonizing the colony stimulating factor-1 receptor on macrophages [57]. It remains to be determined whether such approaches for suppressing macrophage polarization to an M2 state are similarly sufficient to enhance immunological control (presumably by multiple mechanisms) in other cancers and in human patients.

The core HDC modeling approach described here is readily adaptable to the analysis of various TME-associated phenomena. For example, the roles of specific biochemical pathways or signal transduction cascades could be addressed by including these processes explicitly within each agent, potentially allowing for direct comparison with experimental data [64–66]. Additional sources of heterogeneity could also be considered, such as spatial variations in epithelial tissue structure or extracellular matrix (ECM) density [34]. Additional cell types could also be included to address timescales and questions in tumor development beyond those considered here. Because the HDC framework enables specification of cellular behaviors at various levels of detail, it is possible to postulate the behavior of many cell types *in vivo* based upon rigorous *in vitro* observations, and with this goal in mind, such high-dimensional functional characterization experiments may be designed [67]. The HDC framework may also be particularly useful as a design tool for synthetic biology, in which “top-down” engineering of cellular functions starting from an abstract functional specification (e.g., a programmed cellular behavior that appears therapeutically attractive *in silico*) is a prominent goal of the field [40,68–70]. Moreover, HDC model sensitivity analyses, such at those described here, could facilitate enhancing design robustness, for example by identifying cell-based therapy designs that perform well over parameter variations that represent patient-to-patient differences and other practical sources of variability. Thus, subsequent investigations may leverage the findings and approaches described here to both build greater understanding of TME development and to develop therapeutic strategies that modulate tumor-associated immunity to improve cancer treatment.

## Supporting Information

S1 Fig Network of interactions between cellular agent classes via diffusible signals. (A) M1 cells secrete tumor lethality signal (TLS), which induces tumor cell apoptosis when TLS exceeds a specified threshold. (B) Vasculature produces oxygen, which is taken up by tumor cells and all classes of macrophages. All cells die via necrosis without oxygen. (C) Tumor cells secrete Activator, and both the tumor and M2 cells secrete M2S. Naïve macrophages polarize to M1 cells in the presence high Activator and low M2S, and naïve macrophages polarize to M2 cells in the presence of high Activator and high M2S. (D) Naïve macrophages are recruited at a rate proportional to vascularization, which increases with increased M2S. (E) Naïve and M1 macrophages chemotax along gradients of M2S.
(PDF)

S2 Fig Schematic overview of algorithmic details. At each time point, each cell evaluates and contributes to its environment (including both soluble factors and neighboring cells). Thus, the simulation implements each of the 8 steps described on the left, in the order shown. Through these steps, individual cells utilize information obtained from the environment to make decisions (in a stochastic fashion) regarding whether and how to polarize, die, chemotax etc., and thereby, each cell’s respective state and/or location is updated. Complete algorithmic details are provided in S1 Text.
(PDF)

S3 Fig Impact of stochastic macrophage chemotaxis on model behavior. This figure represents a set of simulations and analyses that mirror those presented in Fig. 3A-B; the only difference here is the addition of a stochastic component to the macrophage chemotaxis “rule,” which was previously described [34]. As in Fig. 3, the panels depict the time evolution of cell counts (by type, color-coded to match the key in Fig. 3) for runs in which the tumor survived (A) or died (B). Notably, these panels are essentially identical to those in Fig. 3, demonstrating that adding this stochastic component to macrophage chemtaxis did not impact model behavior.
(PDF)

S4 Fig Spatial predictors of tumor survival. Spatial metrics of the TME at t = 0.5 d, including domain-wide maximum M2S value vs. either mean local coefficient of variation (CV) of M2S (A) or domain-wide mean M2S value (B), with data points indicating individual simulation runs classified by outcome. Local CV of M2S was calculated within each 10 x 10 lattice site (LS) array, and the mean of all 100 such arrays across the domain is shown.
(PDF)

S5 Fig Expanded multi-parameter sensitivity analysis (MPSA). Parameter sensitivity was evaluated using 5 values of each parameter, spanning two orders of magnitude distributed logarithmically, over the range [0.1, 0.3, 1.0, 3.0, 10.0] * p_i_ (where p_i_ is the base case value of each parameter). All pair-wise combinations of parameter values were simulated and 50 simulations were done for each pair of parameter values. Notably, tumor survival was insensitive to parameter values over much of this larger range. In particular, tumor survival essentially became deterministic in response to changes in the diffusion coefficient, rate of MP TLS secretion, initial M2S, tumor TLS threshold, and the MP polarization threshold. However, some novel sensitivities were identified using this larger range. For example, initial tumor size increased survival probability only when very large (250 cells) or very small (3 cells) initial tumors were simulated, indicating that the initial size of the metastatic tumor did not strongly alter survival likelihood except in these extreme cases.
(PDF)

S6 Fig Predictability of tumor survival by MPI for low and high M2 feedback. (A) MPI at t = 0.5 days is plotted against tumor survival probability for only the lowest level of M2 feedback in the MPSA. The correlation with tumor survival in this case is lower than the correlation of tumor survival with MPI across the entire parameter set (Fig. 4D). (B) MPI at t = 0.5 days is plotted against tumor survival probability for only the highest level of M2 feedback in the MPSA. The correlation with tumor survival in this case is higher than that observed across the entire parameter set.
(PDF)

S1 Table Parameter estimation and rationale. Our model contains 18 tunable parameters, base parameter value estimates for which were drawn from the literature or selected to yield qualitative agreement between simulations and experimentally observed phenomena, when appropriate. This table defines and describes each of these parameters, lists the base values used for simulations, and provides relevant source material and justification for the values selected (see accompanying table notes).
(PDF)

S2 Table Core model assumptions and rationale. This table summarizes the core assumptions made in order to develop a model best suited to investigating the specific research questions posed in this study. This table enumerates key assumptions and provides both rationale and citations to the relevant research literature to provide justification for each assumption.
(PDF)

S1 Text Model development and implementation. This documents includes complete details and rationale for all modeling choices including background, model scope and rationale, simulation initiation and propagation, and algorithmic details.
(PDF)

S1 Movie Tumor survives. This movie shows a representative simulation that results in tumor survival under the base parameter set. Tumor cells are shown top left (red: alive, black: dead); Macrophages are shown top middle (blue: naïve, red: M1, green: M2). The four diffusible signals (TLS, M2S, Oxygen, Activator) are shown top right and in the bottom row, respectively. Concentrations are represented by color (low = blue, medium = yellow/green, red = high).
(MP4)

S2 Movie Tumor survives. Same as S1 Movie, but file format: avi.
(AVI)

S3 Movie Tumor dies. This movie shows a representative simulation that results in tumor death under the base parameter set. All cells and signals are the same as in S1 Movie (see caption for S1 Movie).
(MP4)

S4 Movie Tumor dies. Same as S3 Movie, but file format: avi.
(AVI)

S5 Movie Tumor survival with high spatial heterogeneity. This movie shows a representative simulation that results in tumor survival in a regime with high spatial heterogeneity. The first M2 macrophage arrives substantially earlier than in either S1 Movie or S3 Movie, resulting increased M2 feedback and ultimately the survival of the tumor. All cells and signals are the same as in S1 Movie (see caption for S1 Movie).
(MP4)

S6 Movie Tumor survival with high spatial heterogeneity. Same as S5 Movie, but file format: avi.
(AVI)

S7 Movie Tumor survival with high functional heterogeneity. This movie shows a representative simulation that results in tumor survival in a regime with high functional heterogeneity. As in S5 Movie, the presence of cells with high functional heterogeneity ensures the early arrival of M2 macrophages and subsequently increases M2 feedback, which together reinforce the pro-tumor state of the TME and ensure tumor survival. All cells and signals are the same as in S1 Movie (see caption for S1 Movie).
(MP4)

S8 Movie Tumor survival with high functional heterogeneity. Same as S7 Movie, but file format: avi.
(AVI)

S9 Movie Tumor death with high functional heterogeneity low M2 feedback. This movie shows a representative simulation that results in tumor death in a regime with high functional heterogeneity and low secretion of M2S by M2 cells. Although M2 cells appear early in the simulation, with low secretion of M2S, these cells have a minimal effect on the overall state of the TME. In particular, naïve macrophages continue to polarize to M1 cells substantially after the arrival of the first M2 macrophage (which is not the case in S5 Movie and S7 Movie), and the tumor is eventually killed. All cells and signals are the same as in S1 Movie (see caption for S1 Movie).
(MP4)

S10 Movie Tumor death with high functional heterogeneity low M2 feedback. Same as S9 Movie, but file format: avi.
(AVI)
