# Supplementary figures and images for: Spatial and Functional Heterogeneities Shape Collective Behavior of Tumor-Immune Networks
Source: PLoS Comput Biol. 2015 Apr 23;11(4):e1004181. doi: 10.1371/journal.pcbi.1004181 (PMC4408028; doi:10.1371/journal.pcbi.1004181)

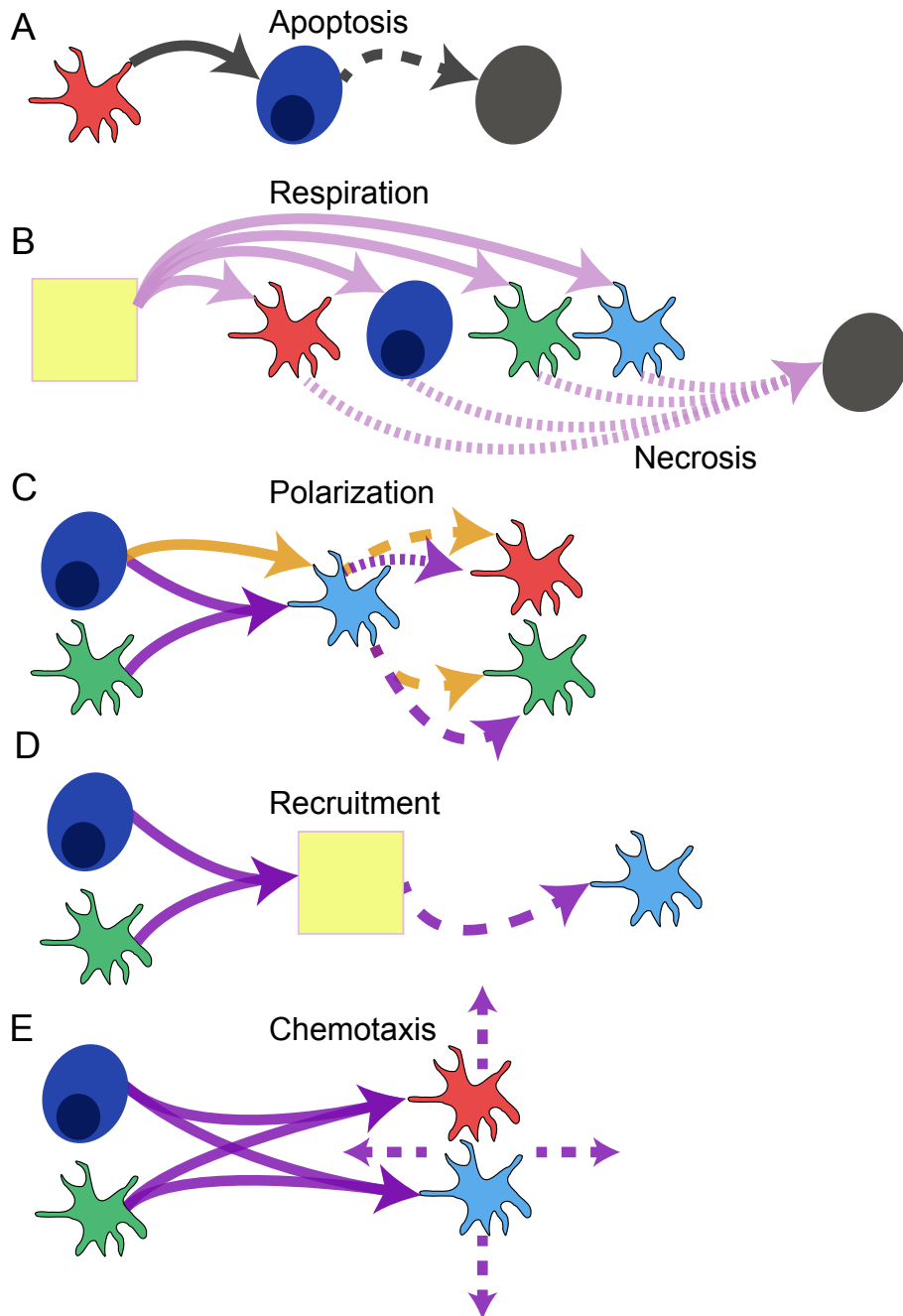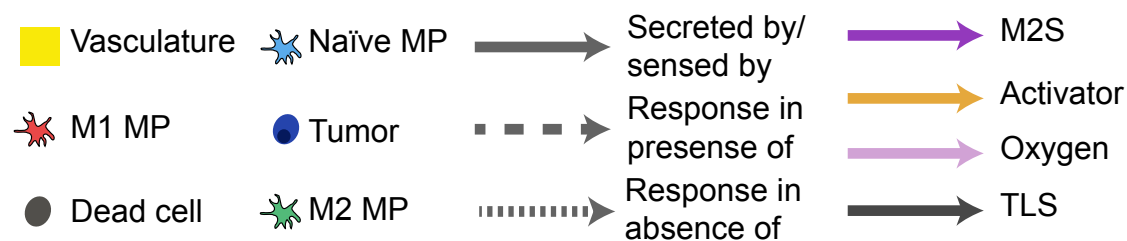

Supplement: S1 Fig — (A) M1 cells secrete tumor lethality signal (TLS), which induces tumor cell apoptosis when TLS exceeds a specified threshold. (B) Vasculature produces oxygen, which is taken up by tumor cells and all classes of macrophages. All cells die via necrosis without oxygen. (C) Tumor cells secrete Activator, and both the tumor and M2 cells secrete M2S. Naïve macrophages polarize to M1 cells in the presence high Activator and low M2S, and naïve macrophages polarize to M2 cells in the presence of high Activator and high M2S. (D) Naïve macrophages are recruited at a rate proportional to vascularization, which increases with increased M2S. (E) Naïve and M1 macrophages chemotax along gradients of M2S. (PDF) [file pcbi.1004181.s001.pdf]

A

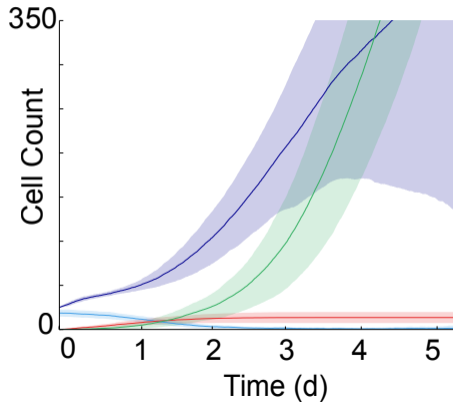

B

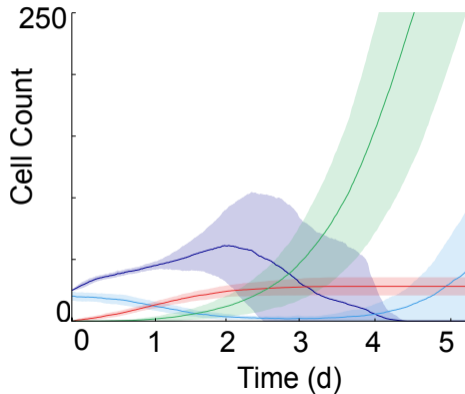

Supplement: S3 Fig — This figure represents a set of simulations and analyses that mirror those presented in Fig. 3A-B; the only difference here is the addition of a stochastic component to the macrophage chemotaxis “rule,” which was previously described [34]. As in Fig. 3, the panels depict the time evolution of cell counts (by type, color-coded to match the key in Fig. 3) for runs in which the tumor survived (A) or died (B). Notably, these panels are essentially identical to those in Fig. 3, demonstrating that adding this stochastic component to macrophage chemtaxis did not impact model behavior. (PDF) [file pcbi.1004181.s003.pdf]

A

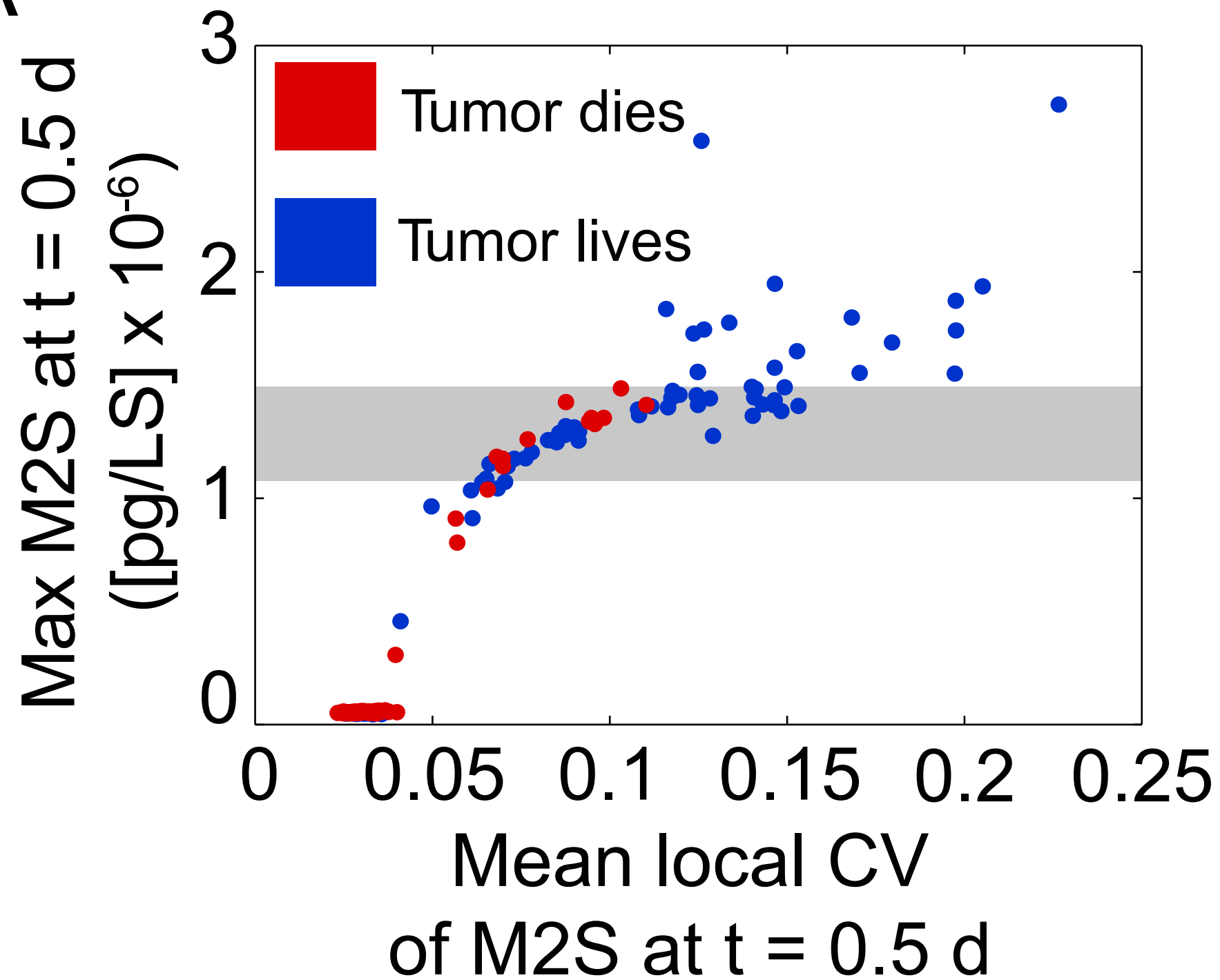

B

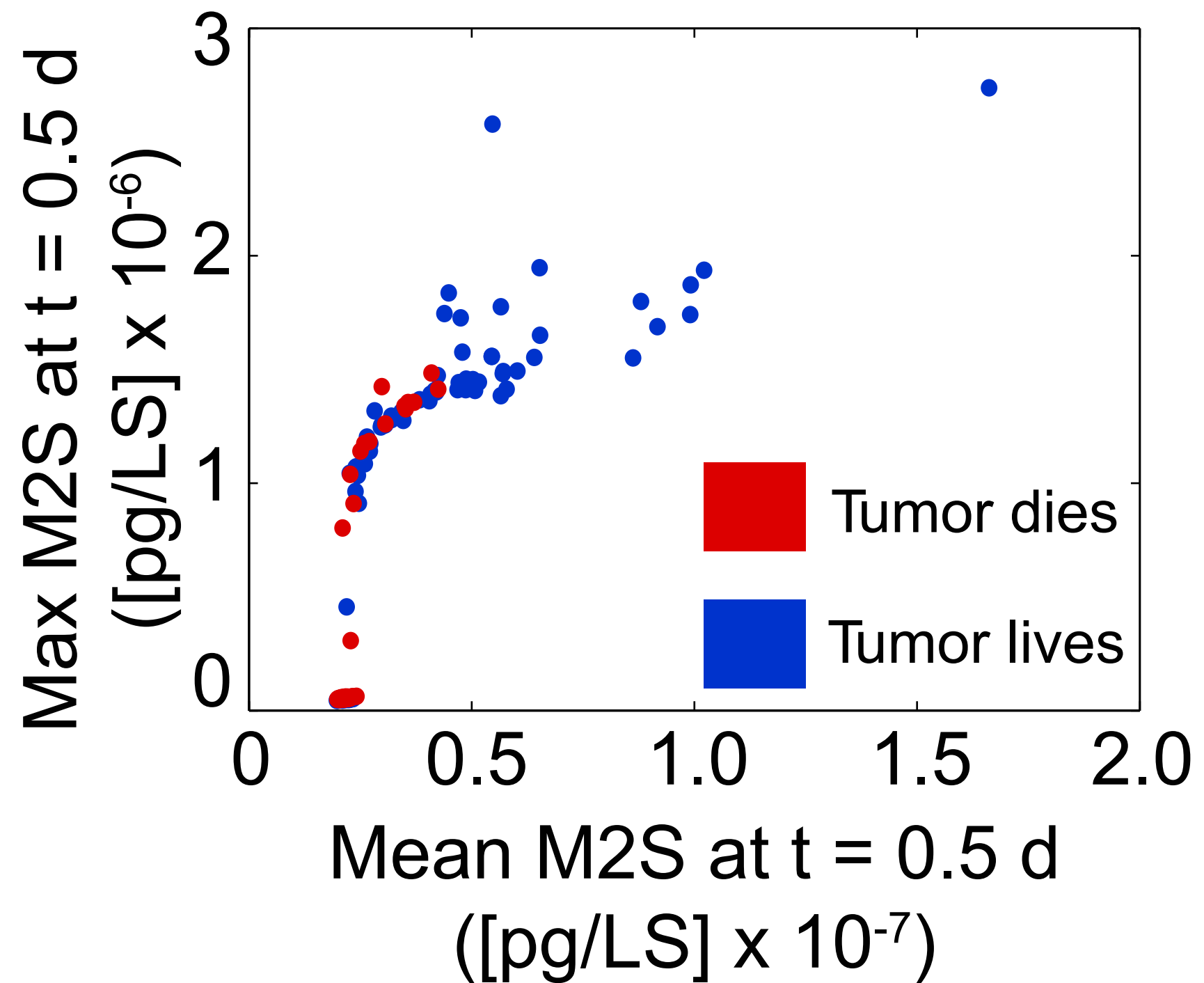

Supplement: S4 Fig — Spatial metrics of the TME at t = 0.5 d, including domain-wide maximum M2S value vs. either mean local coefficient of variation (CV) of M2S (A) or domain-wide mean M2S value (B), with data points indicating individual simulation runs classified by outcome. Local CV of M2S was calculated within each 10 x 10 lattice site (LS) array, and the mean of all 100 such arrays across the domain is shown. (PDF) [file pcbi.1004181.s004.pdf]

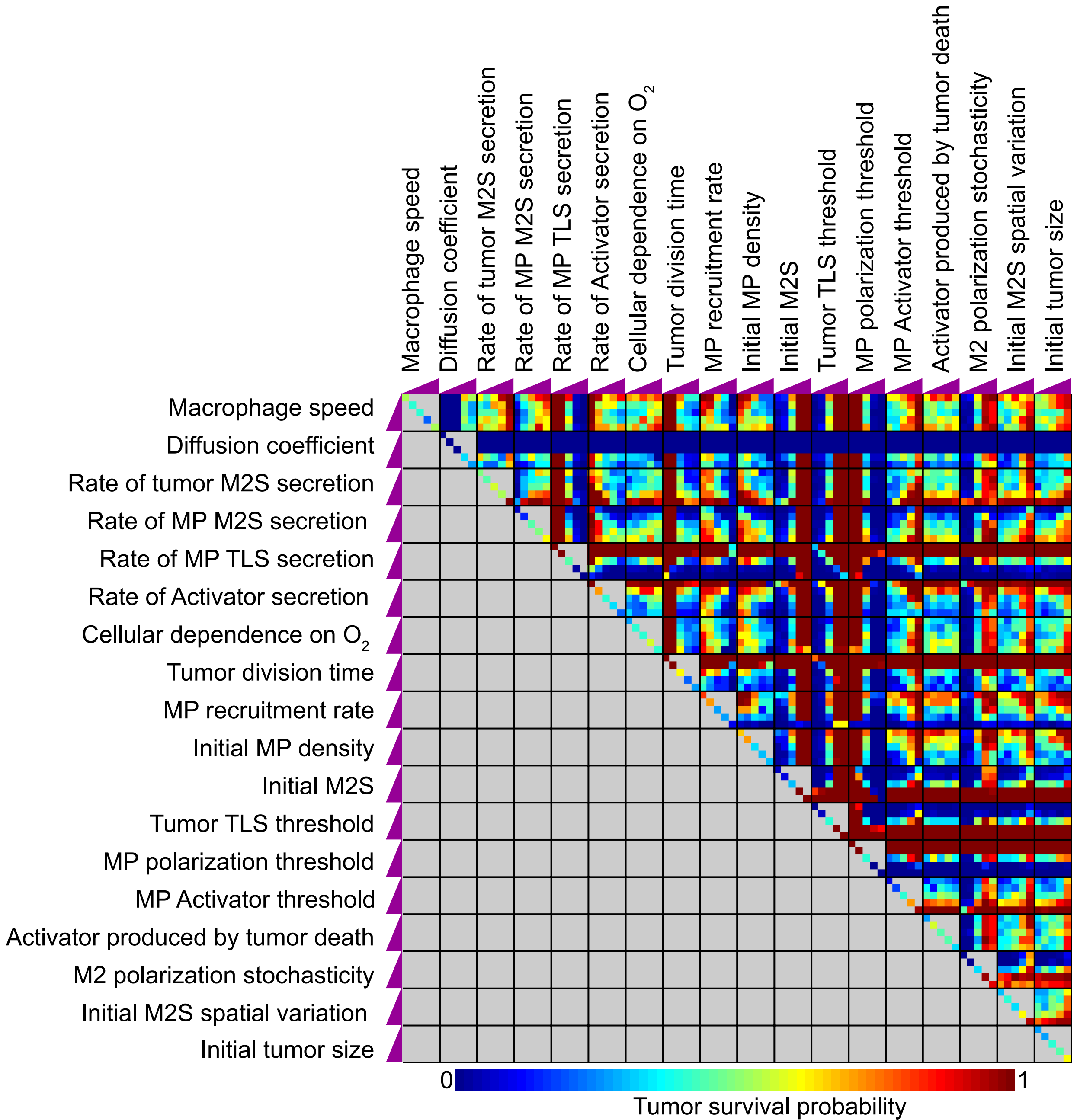

Supplement: S5 Fig — Parameter sensitivity was evaluated using 5 values of each parameter, spanning two orders of magnitude distributed logarithmically, over the range [0.1, 0.3, 1.0, 3.0, 10.0] * pi (where pi is the base case value of each parameter). All pair-wise combinations of parameter values were simulated and 50 simulations were done for each pair of parameter values. Notably, tumor survival was insensitive to parameter values over much of this larger range. In particular, tumor survival essentially became deterministic in response to changes in the diffusion coefficient, rate of MP TLS secretion, initial M2S, tumor TLS threshold, and the MP polarization threshold. However, some novel sensitivities were identified using this larger range. For example, initial tumor size increased survival probability only when very large (250 cells) or very small (3 cells) initial tumors were simulated, indicating that the initial size of the metastatic tumor did not strongly alter survival likelihood except in these extreme cases. (PDF) [file pcbi.1004181.s005.pdf]

**A**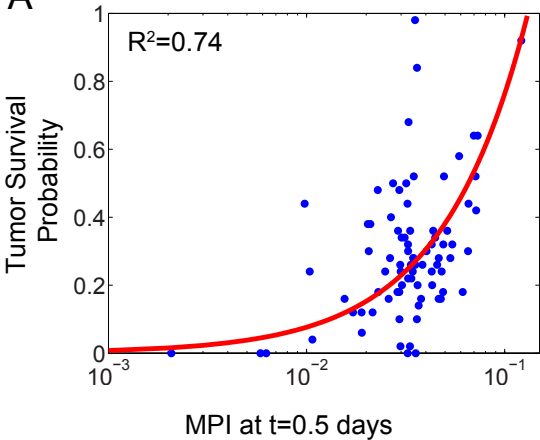**B**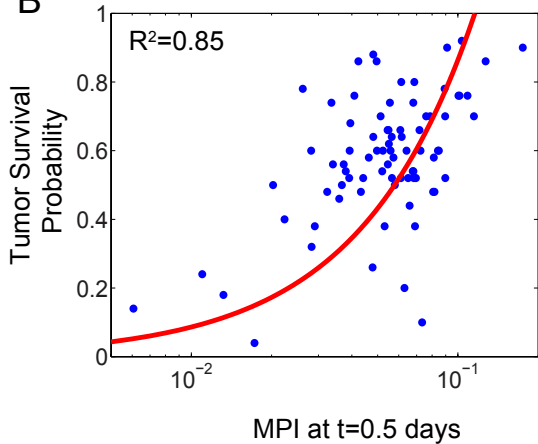

Supplement: S6 Fig — (A) MPI at t = 0.5 days is plotted against tumor survival probability for only the lowest level of M2 feedback in the MPSA. The correlation with tumor survival in this case is lower than the correlation of tumor survival with MPI across the entire parameter set (Fig. 4D). (B) MPI at t = 0.5 days is plotted against tumor survival probability for only the highest level of M2 feedback in the MPSA. The correlation with tumor survival in this case is higher than that observed across the entire parameter set. (PDF) [file pcbi.1004181.s006.pdf]
