# Supplementary material for: Spatial and Functional Heterogeneities Shape Collective Behavior of Tumor-Immune Networks
Source: PLoS Comput Biol. 2015 Apr 23;11(4):e1004181. doi: 10.1371/journal.pcbi.1004181 (PMC4408028; doi:10.1371/journal.pcbi.1004181)
Supplement: S2 Fig — At each time point, each cell evaluates and contributes to its environment (including both soluble factors and neighboring cells). Thus, the simulation implements each of the 8 steps described on the left, in the order shown. Through these steps, individual cells utilize information obtained from the environment to make decisions (in a stochastic fashion) regarding whether and how to polarize, die, chemotax etc., and thereby, each cell’s respective state and/or location is updated. Complete algorithmic details are provided in S1 Text. (PDF) [file pcbi.1004181.s002.pdf]

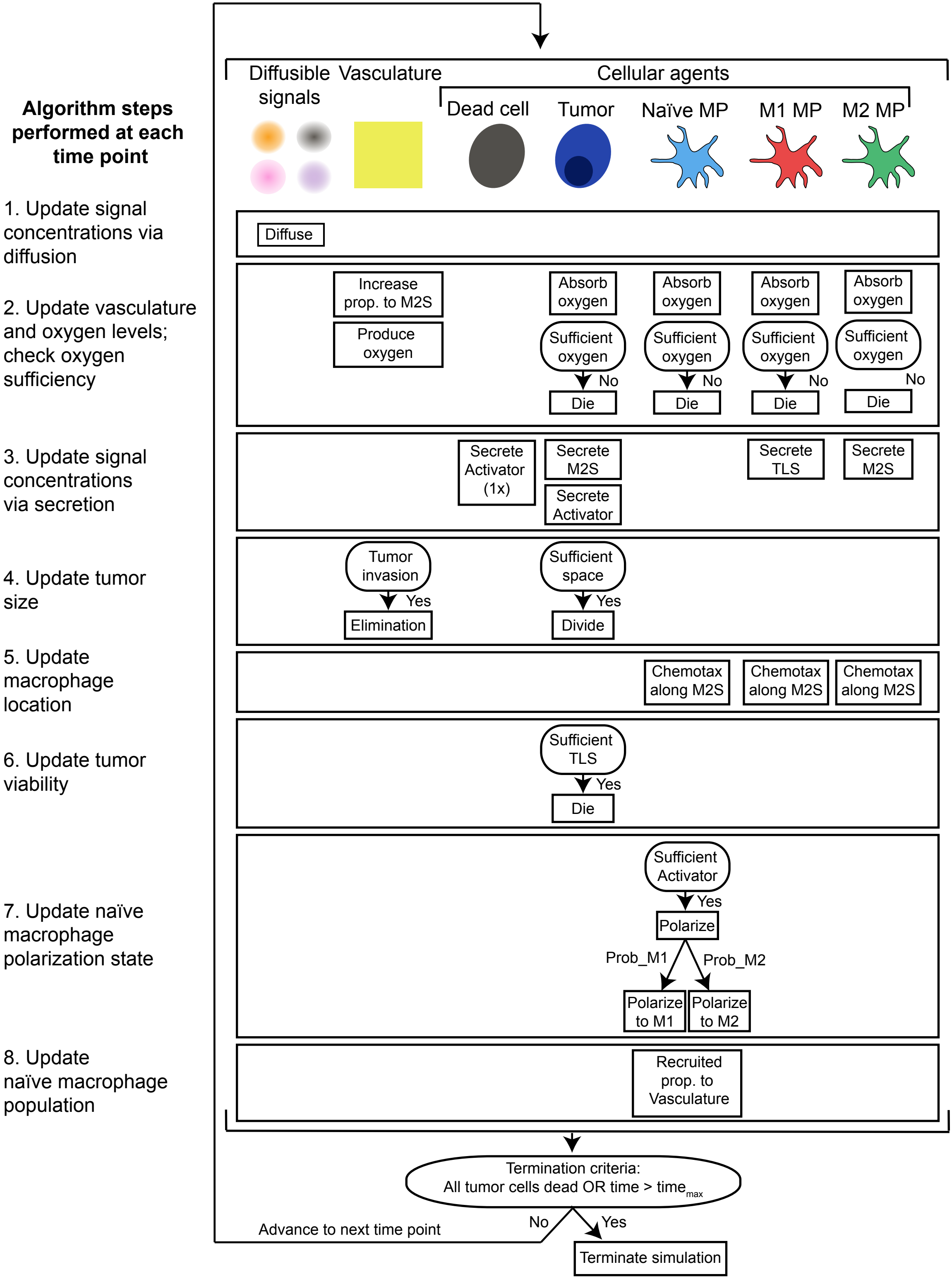

Algorithm steps performed at each time point
1. Update signal concentrations via diffusion
2. Update vasculature and oxygen levels; check oxygen sufficiency
3. Update signal concentrations via secretion
4. Update tumor size
5. Update macrophage location
6. Update tumor viability
7. Update naïve macrophage polarization state
8. Update naïve macrophage population
Diffusible signals
Vasculature
Cellular agents
Dead cell
Tumor
Naïve MP
M1 MP
M2 MP
Diffuse
Increase prop. to M2S
Produce oxygen
Absorb oxygen
Sufficient oxygen
No
Die
Secrete Activator (1x)
Secrete M2S
Secrete Activator
Secrete TLS
Secrete M2S
Tumor invasion
Yes
Elimination
Sufficient space
Yes
Divide
Chemotax along M2S
Sufficient TLS
Yes
Die
Sufficient Activator
Yes
Polarize
Prob_M1
Prob_M2
Polarize to M1
Polarize to M2
Recruited prop. to Vasculature
Termination criteria:
All tumor cells dead OR time > time$_{max}$
No
Yes
Advance to next time point
Terminate simulation
