# Supplementary material for: Spatial and Functional Heterogeneities Shape Collective Behavior of Tumor-Immune Networks
Source: PLoS Comput Biol. 2015 Apr 23;11(4):e1004181. doi: 10.1371/journal.pcbi.1004181 (PMC4408028; doi:10.1371/journal.pcbi.1004181)
Supplement: S1 Table — Our model contains 18 tunable parameters, base parameter value estimates for which were drawn from the literature or selected to yield qualitative agreement between simulations and experimentally observed phenomena, when appropriate. This table defines and describes each of these parameters, lists the base values used for simulations, and provides relevant source material and justification for the values selected (see accompanying table notes). (PDF) [file pcbi.1004181.s007.pdf]

Table S1. Parameter estimation and rationale

Name	Symbol	Value in Base Case	Notes
Macrophage speed	*p1*	0.01 $\frac{\mu m}{sec}$	(1)
Diffusion coefficients	*p2*	30 $\frac{\mu m^2}{sec}$	(2)
Diffusion coefficient of O_2	*p2*	300 $\frac{\mu m^2}{sec}$	(3)
Rate of M2 secretion by tumor	*p3*	$1 \times 10^{-11} \frac{pg}{cell \times sec}$	(4)
Rate of M2 secretion by M2 cells	*p4*	$6 \times 10^{-9} \frac{pg}{cell \times sec}$	(5)
Rate of tumor lethality signal secretion by M1 cells	*p5*	$4.5 \times 10^{-9} \frac{pg}{cell \times sec}$	(6)
Rate of Activator signal secretion by tumor cells	*p6*	$6 \times 10^{-8} \frac{pg}{cell \times sec}$	(7)
Oxygen uptake ratio	*p7*	100 (dimensionless)	(8)
Tumor division time	*p8*	18 hours	(9)
Macrophage recruitment rate	*p9*	$1 \times 10^{-8} \frac{cells}{lattice\ site \times sec \times vasculature}$	(10)
Initial macrophage density	*p10*	$2 \times 10^{-3} \frac{cells}{lattice\ site}$	(11)
Initial M2 signal concentration	*p11*	$2 \times 10^{-8} \frac{pg}{lattice\ site}$	(12)
Tumor threshold of tumor lethality signal	*p12*	$2 \times 10^{-6} \frac{pg}{lattice\ site}$	(13)
Macrophage threshold for M2 signal	*p13*	$4 \times 10^{-8} \frac{pg}{lattice\ site}$	(14)
Naïve macrophage polarization threshold of Activator signal	*p14*	$8 \times 10^{-6} \frac{pg}{lattice\ site}$	(15)
Activator signal produced by tumor death	*p15*	$6 \times 10^{-7} \frac{pg}{cell \times sec}$	(16)
Macrophage polarization stochasticity<br><br>("Functional heterogeneity")	*p16*	1 (dimensionless)	(17)
Initial M2S spatial variation<br><br>("Spatial heterogeneity")	*p17*	$5 \times 10^{4} \frac{pg}{lattice\ site}$	(18)
Initial tumor size	*p18*	25 cells	(19)

Table S1 Notes

Parameters estimated from experimental measurements

Base values for the following parameters were estimated based upon direct comparisons with previously reported experimental measurements.

1. Macrophage chemotaxis rate
The speed of macrophage chemotaxis is a rate-of-travel parameter. Because space is discretized, macrophages cannot move continuously, and thus this parameter represents the average speed of macrophage chemotaxis. The value selected for this parameter was based upon an investigation in which macrophage chemotaxis speed was measured in the presence of CSF-1 [1].

2. Diffusion
Diffusion is one of the main processes by which soluble factors are transported throughout the cell. To simplify our simulations, we approximate the diffusion constant for each of the three secreted factors (M2 signal, Activator signal, and tumor lethality signal) in tissue as being approximately 1/3 that of green fluorescent protein in water, which was measured by correlation fluorescence microscopy [2]. This is a reasonable approximation because the "lumped" factors above represent actual proteins (e.g., IL-10, HMGB1, TNFα), all of which have a molecular weight that is within an order of magnitude of the weight of GFP, and the apparent diffusion coefficient in human tissue is generally measured to be between 1/2 to 1/3 of the value measured in water [3].

3. Diffusion of oxygen
Soluble oxygen is much smaller than all of the diffusible proteins discussed above and in general will diffuse faster than proteins. As such, we approximate the diffusion of oxygen as occurring 10 times faster than protein factors. Importantly, this value is set to *always* be 10 times the diffusion constant of the soluble protein factors, effectively making this parameter a function of *p2* (as indicated in Table S1). The base value used for the oxygen diffusion rate is within an order of magnitude of the estimated value for the diffusion of oxygen in tissue [4].

4. Secretion of M2 signal by the tumor
The secretion rate of M2 signal by the tumor was estimated based upon the secretion rate of IL-10 by a tumor cell line (B16 melanoma cell) using ELISA [5].

5. Secretion of M2 signal by M2 cells.
The secretion rate of M2 signal by M2 cells was estimated based upon the secretion rate of IL-10 by M2 macrophages, which was measured by ELISA [6].

6. Secretion of tumor lethality signal by M1 macrophages.
The secretion rate of tumor lethality signal by M1 cells was estimated based upon the secretion rate of TNFα by M1 macrophages, which was measured following stimulation with amyloidβ [7].

7. Secretion of Activator signal by tumor cells.
The secretion of Activator signal by tumor cells was estimated based upon the secretion rate of high mobility group box 1 (HMGB1) by tumor cells (human mesothelioma cells) [8].

8. Oxygen consumption ratio
Oxygen is both consumed and released into this system at a very high rate. To simplify our parameterization of this process, we use only a single, non-dimensional ratio that measures how much faster oxygen is consumed compared to how fast it is released, at the basal level of vascularization. The base value of this ratio was set so that approximately 50% of the tumor would be necrotic without additional macrophage influx/vascularization. All cells are estimated to consume oxygen at a rate of 8×10^{-4} pg/(cell sec), based upon direct measurements [9].

9. Tumor division time
Tumor cell division time was estimated based upon the division time of HeLa cells in culture (representing aggressive growth), which is similar to other cells lines and has been measured as approximately 20 hours [10].

19. Initial tumor size
Distal metastases are assumed to begin as a small cluster of cells originating from circulating tumor cells (CTCs) [11]. In accordance with experimental estimates measuring CTC size in prostate cancer, we estimate the average initial tumor size to be 25 cells [12].

Parameters estimated using indirect measurements, approximations, or phenomena

Base values for the following parameters were estimated or approximated indirectly using reported experimental values, and when necessary, were selected to recapitulate known biological phenomena with model behavior.

10. Macrophage recruitment rate
This parameter sets the rate at which macrophages are recruited to individual lattice sites and is proportional to the vascularization at that site. At the tumor site, macrophages occupy between 1% to 10% of the surrounding tumor microenvironment (TME) [13]. To account for this, we selected the base value of the macrophage recruitment rate such that on average, at the base values of other parameters, the macrophage population would grow to cover approximately 3 - 4% of available space surrounding the tumor.

11. Initial macrophage density
This parameter sets the initial average density of naïve macrophages throughout the TME. The base value of this parameter was selected so that there would be approximately the same number of immune cells as tumor cells (approximately 20) at the start of each simulation, which we deemed to be the most reasonable and conservative assumption to be made in the absence of evidence that one or the other population is substantially larger at the time of implantation.

12. Initial M2 signal level
The parameter sets the average amount of M2 signal at each lattice site at the start of each simulation. The base value was estimated (approximately) using the concentration of IL-10 in the serum of healthy humans [14].

13. Tumor threshold to tumor lethality signal
This parameter sets the level of tumor lethality signal above which the tumor will die. This value was set such that tumor survival probability was approximately 0.5 at the base values of all other parameters, enabling us to evaluate model behavior in a regime that is not dominated by (trivial) tumor death or tumor survival, and recapitulating the expected workings of partial control of new tumors by immune surveillance [15].

14. Macrophage polarization threshold
This parameter sets the level of M2 signal above which naïve macrophages, in the presence of high enough levels of Activator signal, will polarize to M2 (when polarization is deterministic – see Note 18 for the case of stochastic polarization). We set the base value of this parameter (*p13*) to be twice that of the base value of the initial M2 signal (*p11*), reasoning that a 2x increase in cytokine concentration over the basal level may comprise a reasonable lower bound to the fold-change in an environmental signal required to induce a cellular response. Note that aside from setting base values, the value of *p13* is independent of the value of *p11*.

15. Naive macrophage polarization threshold for Activator signal
This parameter sets the concentration of Activator signal above which a naïve macrophage will become activated and polarize to either an M1 or M2 state, depending on the local level of M2 signal. We estimated this value based upon the concentration of lipopolysaccharide (LPS) needed to activate macrophages via TLR4 [16], which is also the receptor responsive to HMGB1 [8].

16. Activator produced upon tumor cell death via necrosis
Cells that die via necrosis release macrophage-activating signals including HMGB1 [17]. In a study comparing the secretion rate of HMGB1 by apoptotic and necrotic cancer cells, the latter released approximately 10 times as much HMGB1 as the former, when measured via ELISA [18]. To capture this effect in the model, the base value of this parameter was set at 10 times the base value of the secretion level of HMGB1 by tumor cells (*p6*). Note that aside from setting base values, the value of *p15* is independent of the value of *p6*.

Heterogeneity parameters

These parameters tune of the amount of functional and spatial heterogeneity (respectively) in our model. Because they are more phenomenological than the other parameters in our model, these parameters were tuned independently to enable robust characterization of the qualitative behaviors of the model.

17. Stochastic macrophage polarization ("Functional heterogeneity")
This parameter tunes the functional heterogeneity in the TME by altering the probability that, for a given ratio of M2 signal to M2 polarization threshold, an activated macrophage will polarize to the M2 state. The base value for this parameter was set such that when the ratio of initial M2 signal to M2 polarization threshold (*p11/p13*) is equal to 1 (the lowest value of *p11* where polarization to an M2 state would occur universally, in the absence of spatial and functional heterogeneities), the probability of polarization to the M2 state is 50%.

18. Initial M2S spatial variation ("Spatial heterogeneity")
This parameter tunes spatial heterogeneity in the TME by altering the variance of the spatial distribution of the initial M2 signal. Specifically, to initialize the M2S array at the start of a simulation, a random normal number with mean *p11* and variance *p17* is chosen at each lattice site. This grainy distribution is smoothed out almost immediately from diffusion. This parameter was set so that when the model was run using base parameter values, on average there would be pockets of M2 signal above the M2 threshold (*p13*) at the time when macrophages begin to activate (around 2.5-3 h after simulation initiation).

References Cited in Table S1

1. Allen WE (1998) A Role for Cdc42 in Macrophage Chemotaxis. J Cell Biol 141: 1147-1157.
2. Terry BR, Matthews EK, Haseloff J (1995) Molecular Characterization of Recombinant Green Fluoresecnt Protein by Fluoresence Correlation Microscopy. Biochemical and Biophysical Research Communications 217: 21-27.
3. Sener RN (2001) Diffusion MRI: apparent diffusion coefficient (ADC) values in the normal brain and a classification of brain disorders based on ADC values. Computerized Medical Imaging and Graphics 25: 299-326.
4. Evans NTS, Naylor PFD, Quinton TH (1981) The Diffusion Coefficient of Oxygen in Respiring Kidney and Tumor Tissue. Respiration Physiology 43: 179-188.
5. Garcia-Hernandez ML, Hernandez-Pando R, Gariglio P, Berumen J (2002) Interleukin-10 promotes B16-melanoma growth by inhibition of macrophage functions and induction of tumour and vascular cell proliferation. Immunology 105: 231-243.
6. Edwards JP, Zhang X, Frauwirth KA, Mosser DM (2006) Biochemical and functional characterization of three activated macrophage populations. J Leukoc Biol 80: 1298-1307.
7. Fiala M, Zhang L, Gan XH, Sherry B, Taub D, et al. (1998) Amyloid-beta induces chemokine secretion and monocyte migration across a human blood-brain barrier model. Molecular Medicine 4: 480-489.
8. Jube S, Rivera ZS, Bianchi ME, Powers A, Wang E, et al. (2012) Cancer cell secretion of the DAMP protein HMGB1 supports progression in malignant mesothelioma. Cancer Research 72: 3290-3301.
9. Pilatus U, Aboagye E, Artemov D, Mori N, Ackerstaff E, et al. (2001) Real-time measurements of cellular oxygen consumption, pH, and energy metabolism using nuclear magnetic resonance spectroscopy. Magn Reson Med 45: 749-755.

10. Puck TT, Marcus PI, Cieciura SJ (1956) Clonal growth of mammalian cells invitro - growth characteristics of colonies from single HeLa cells with and without a feeder layer. Journal of Experimental Medicine 103: 273-&.

11. Chaffer CL, Weinberg RA (2011) A perspective on cancer cell metastasis. Science 331: 1559-1564.

12. Stott SL, Lee RJ, Nagrath S, Yu M, Miyamoto DT, et al. (2010) Isolation and characterization of circulating tumor cells from patients with localized and metastatic prostate cancer. Sci Transl Med 2: 25ra23.

13. Ma J, Liu L, Che G, Yu N, Dai F, et al. (2010) The M1 form of tumor-associated macrophages in non-small cell lung cancer is positively associated with survival time. Bmc Cancer 10.

14. Lopatin U, Yao X, Williams RK, Bleesing JJH, Dale JK, et al. (2001) Increases in circulating and lymphoid tissue interleukin-10 in autoimmune lymphoproliferative syndrome are associated with disease expression. Blood 97: 3161-3170.

15. Dunn GP, Bruce AT, Ikeda H, Old LJ, Schreiber RD (2002) Cancer immunoediting: from immunosurveillance to tumor escape. Nat Immunol 3: 991-998.

16. Meng FY, Lowell CA (1997) Lipopolysaccharide (LPS)-induced macrophage activation and signal transduction in the absence of Src-family kinases Hck, Fgr, and Lyn. Journal of Experimental Medicine 185: 1661-1670.

17. Degryse B, Bonaldi T, Scaffidi P, Muller S, Resnati M, et al. (2001) The high mobility group (HMG) boxes of the nuclear protein HMG1 induce chemotaxis and cytoskeleton reorganization in rat smooth muscle cells. Journal of Cell Biology 152: 1197-1206.

18. Luo Y, Chihara Y, Fujimoto K, Sasahira T, Kuwada M, et al. (2013) High mobility group box 1 released from necrotic cells enhances regrowth and metastasis of cancer cells that have survived chemotherapy. European Journal of Cancer 49: 741-751.
