# Supplementary material for: Spatial and Functional Heterogeneities Shape Collective Behavior of Tumor-Immune Networks
Source: PLoS Comput Biol. 2015 Apr 23;11(4):e1004181. doi: 10.1371/journal.pcbi.1004181 (PMC4408028; doi:10.1371/journal.pcbi.1004181)
Supplement: S2 Table — This table summarizes the core assumptions made in order to develop a model best suited to investigating the specific research questions posed in this study. This table enumerates key assumptions and provides both rationale and citations to the relevant research literature to provide justification for each assumption. (PDF) [file pcbi.1004181.s008.pdf]

Table S2. Core model assumptions with justification

| Core Model Assumption | Rationale/Reference |
|---|---|
| Tumor cells do not evolve. | Our model endeavors to describe the establishment of novel tumors following metastasis of cancer cells that are already invasive (including the capacity to degrade extracellular matrix, for example by secretion of matrix metalloproteinases). Additional tumor cell evolution was assumed to require time scales longer than the time spans being simulated (~5 days). Thus, each simulation considers a single invasive tumor cell phenotype. |
| All tumor cells are constrained equally by surrounding epithelial cells and thus divide at the same rate throughout the simulation. | As described above, we do not consider evolution of invasiveness over the timescale being simulated, and all tumor cells are assumed to be equally invasive. Along with this, because we are most interested in understanding the role of immunological heterogeneity in tumor survival, we do not include heterogeneity of ECM or surrounding epithelia in our model, as done in previous work [1-3]. Due to these assumptions we can describe tumor cell division with a single parameter (the tumor cell division time) that captures the effects of both matrix rigidity and cell division. |
| Naïve macrophages can functionally polarize to one of two states: M1 (tumor suppressing); M2 (tumor promoting). | Although macrophage polarization represents a continuum of functional states, we discretize this continuum into a single M1 and a single M2 state in order to consider phenotypic extremes that are most associated with tumor-associated immune responses. M1 macrophages have the capability of inducing protective inflammation against cancer cells. On the other side of the spectrum, M2 macrophages have been shown to promote tumor growth [4-6]. Specifically, in our study, we highlighted the M2c phenotype that was polarized by IL-10 exposure, due to the role of IL-10 in immunosuppressive environments [4,6]. |
| Macrophage polarization depends only an "Activator" molecule secreted by the tumor (e.g., HMGB1) and an M2 inducer signal (e.g. IL-10) secreted by the tumor and M2 cells. | Previous experiments have demonstrated that macrophages will not functionally polarize in the presence of cytokines known to induce polarization *in vivo* (e.g., IL-10) without the presence of a secondary "Activator" signal, e.g., LPS or HMGB1 [7]. We model this relationship by requiring a threshold of "Activator" be present for polarization of naïve macrophages to occur, and a threshold of M2 inducer (M2S) be present for macrophages to polarize to the M2 state. |

| | |
|---|---|
| M2 macrophages reinforce their own lineage strongly. | M2-like macrophages secrete IL-10, a known M2-inducer [4-6]. |
| All cell types die from anoxia conditions. | It is well known that all cell types will die in the absence of oxygen. |
| Upon death tumor cells will secrete a large dose of macrophage Activator. | Tumor cells secrete HMGB1, a macrophage activator, upon death [8]. |
| Increased vasculature will develop in response to VEGF, which is secreted by tumor cells and M2 cells. | VEGF is shown to be angiogenic and to be secreted by both tumor cells and M2 macrophages [5]. |
| Vasculature does not exist within the tumor. | It is known that the interiors of fast growing, aggressive tumors are poorly vascularized [9]. To capture this effect in our model we assumed that the growing tumor replaces or destroys all vasculature in the spots it expands into, ensuring a hypoxic tumor interior. |
| Initial tumor is small and genetically homogeneous. | We assume that the initial tumor is the result of an implantation event at a site distal from the primary tumor. Such events are typically assumed to involve a small cluster (1-100) of cells [10]. Since this cluster is small we assume it to be genetically homogeneous. We consider the effects of different initial tumor sizes in the MPSA analysis. |
| Secreted VEGF (represented via the lumped mediator M2S) will not generate substantially new vascular architecture. | We assume that the tumor-adjacent tissue possesses some vasculature prior to the initiation of the simulation, and that substantial vasculature remodeling will not occur during the simulation time frame (~5 days). Thus, we assume that the effect of VEGF would be primarily to enhance blood supply via the existing vascular and we do not explicitly describe spatial changes in blood supply due to remodeling of the vasculature. |
| All secreted proteins diffuse at the same rate, and oxygen diffuses 10x faster than do proteins. We consider only changes to diffusion rates that equally impact all diffusible species. | M2S (IL-10, VEGF), TLS (TNFα) and Activator (HMGB1) proteins all have similar molecular weights, and oxygen is much smaller; see Table S1 for a justification of specific numerical values used. We assume that the primary biological feature that would impact diffusion of all species is tissue density, and thus we assume that changing the single model parameter describing diffusion rates would impact all diffusible species in a manner that maintains the relative rates. |
| New macrophages are recruited proportional to the vascularization. | Increased vascularization increases the flow of blood to a lattice site, and since macrophages and macrophage precursors such as monocytes (which are not explicitly considered in this model) may enter a tumor site via the blood, we assume that increased vascularization would result in increased rates of macrophage infiltration. |

| | |
|---|---|
| Degradation of all diffusible factors and uptake of diffusible proteins (M2S, TLS, Activator) is not explicitly represented in the model. | In our simulations, these diffusible proteins diffuse out of TME in approximately 2 hours, which is much shorter than the half-life of the proteins considered, such that degradation is not expected to impact the outcomes of our analyses. Active uptake of the three diffusible proteins was not included explicitly in the model because the cellular response to these molecules is assumed to occur in a way that does not alter their overall mean-field concentrations. Active uptake of oxygen was included in our model, however, since oxygen is known to be a limiting resource within the TME that is also actively taken up by all cell types. |
| Macrophages chemotax deterministically. | Macrophages are known to be highly motile cells that move relatively quickly. Thus, although on a short time scale movement of macrophages may have a stochastic component to it, on the time scales considered in our simulation and in the presence of a suitably strong gradient of M2S, their net movement would be effectively deterministic. |
| A 2-D lattice can capture general features 3-D dynamics. | As in previous models [1], the use of a 2-dimensional lattice is assumed to represent a slice through a 3-dimensional piece of cancerous tissue. While 3-dimensional models can provide more accurate or patient-specific predictions, a number of previously developed 2-d lattice models have provided useful insights in cancer development [1-3]. Examples of where a 2-D model was used to make verifiable, experimental predictions include:<br>1. [1] used a 2-D lattice model to predict that more invasive phenotypes would evolve in more heterogeneous microenvironment.<br>2. [2] used a 2-D lattice model to predict that TGF-β does not have a significant role in tumor survival, which was later experimentally validated.<br>3. [3] used a 2-D lattice model to predict that increased heterogeneity or rigidity in the ECM surrounding a tumor would generate a less isotropic tumor morphology.<br><br>Thus, while such 2-D models may not replicate the *in vivo* environment with complete accuracy, they can successfully be used to make experimentally verifiable predictions about tumor growth and development. |

**References cited in Table S2**

1. Anderson AR, Weaver AM, Cummings PT, Quaranta V (2006) Tumor morphology and phenotypic evolution driven by selective pressure from the microenvironment. Cell 127: 905-915.

2. Basanta D, Strand DW, Lukner RB, Franco OE, Cliffel DE, et al. (2009) The role of transforming growth factor-beta-mediated tumor-stroma interactions in prostate cancer progression: an integrative approach. Cancer Res 69: 7111-7120.

3. Jiao Y, Torquato S (2011) Emergent behaviors from a cellular automaton model for invasive tumor growth in heterogeneous microenvironments. PLoS Comput Biol 7: e1002314.

4. Mantovani A, Sica A, Sozzani S, Allavena P, Vecchi A, et al. (2004) The chemokine system in diverse forms of macrophage activation and polarization. Trends Immunol 25: 677-686.

5. Sica A, Larghi P, Mancino A, Rubino L, Porta C, et al. (2008) Macrophage polarization in tumour progression. Semin Cancer Biol 18: 349-355.

6. Sica A, Bronte V (2007) Altered macrophage differentiation and immune dysfunction in tumor development. J Clin Invest 117: 1155-1166.

7. Stout RD, Jiang C, Matta B, Tietzel I, Watkins SK, et al. (2005) Macrophages sequentially change their functional phenotype in response to changes in microenvironmental influences. J Immunol 175: 342-349.

8. Karin M, Greten FR (2005) NF-kappaB: linking inflammation and immunity to cancer development and progression. Nat Rev Immunol 5: 749-759.

9. Folkman J (1972) Anti-angiogenesis: new concept for therapy of solid tumors. Ann Surg 175: 409-416.

10. Chaffer CL, Weinberg RA (2011) A perspective on cancer cell metastasis. Science 331: 1559-1564.
