# Supplementary material for: Spatial and Functional Heterogeneities Shape Collective Behavior of Tumor-Immune Networks
Source: PLoS Comput Biol. 2015 Apr 23;11(4):e1004181. doi: 10.1371/journal.pcbi.1004181 (PMC4408028; doi:10.1371/journal.pcbi.1004181)
Supplement: S1 Text — This documents includes complete details and rationale for all modeling choices including background, model scope and rationale, simulation initiation and propagation, and algorithmic details. (PDF) [file pcbi.1004181.s009.pdf]

Text S1. Model development and implementation

1. Background Page 2

2. Model scope and rationale Page 3

3. Simulation initiation and propagation Page 8

4. Algorithmic details Page 10

5. References cited in Text S1 Page 12

1. Background

In this study we sought to understand how the tumor microenvironment (TME) evolves over time at the earliest stages of tumor formation, such as occurs following metastasis to a novel site or implantation of a tumor-forming cell line in an animal model of cancer. Given the present challenges of studying this system in vivo, we developed a hybrid discrete-continuous agent based model to enable incorporation of salient spatial features and cellular behaviors. Importantly, this approach enables one to embed rules governing cellular decision-making based upon experimentally observed or proposed behaviors, even if the precise mechanisms underlying such behaviors are unknown. For example, it is known that macrophages chemotax along gradients of specific chemokines secreted by cells in the TME. In an agent-based framework like that used here, we can encode this observation in the form of a rule (e.g. “macrophages travel in the direction of increasing chemokine concentration”), instead of explicitly modeling the full complexity of the receptors, signals transductions pathways, transcription factors, genes, and secretory mechanisms involved. In this way, the core input/output relationship of any particular phenomenon can be captured while adding minimally to the complexity of the model, since complexity adds computational load, necessitates the introduction of extensive extra parameters and assumptions, and ultimately complicates model evaluation and interpretation. Moreover, this rule-based representation enables the incorporation of experimental observations of cell behavior for which underlying molecular mechanisms have not yet been elucidated. Such a rule-based (also termed phenomenological) modeling approach has been utilized and validated extensively to investigate a range of biological phenomena, especially in the context of cancer [1-5].

2. Model scope and rationale

Our general approach was to develop the simplest, minimally parameterized model that adequately captures core features of the specific case of a novel tumor initiated by the implantation of a small, clonal cluster of highly invasive metastatic tumor cells in normal, pre-existing tissue. Key processes represented included vascularization, oxygen uptake, angiogenesis, and macrophage chemotaxis, polarization, activation, and tumor-killing. Biological complexity was reduced by "lumping" functionally related molecules, which are often co-regulated and co-expressed, into single representative soluble factors. For example, the generic "M2 signal" (M2S) represented pro-angiogenic functions mediated by vascular endothelial growth factor (VEGF), pro-M2 functions mediated by interleukin 10 (IL-10), and chemotactic functions mediated by various chemokines, each of which is secreted by tumor cells and M2-type or tumor-associated macrophages [6-8]. Similarly, the "tumor lethality signal" (TLS) secreted by M1 cells most directly represents tumor necrosis factor (TNF), but it may also capture related, co-regulated mechanisms by which M1 cells promote tumor killing [9]. Cellular states were also reduced in complexity – for example, although macrophage polarization likely represents a continuum of states, here these states were discretized. Thus to capture core phenomena, the model contained four diffusible signals and six classes of cellular agent. The algorithm for our HDC approach is outlined in Figure S2 and described in explicit, step-by-step detail in Subsection *4. Algorithmic details.*

Given the specific scenario considered in our model, we did not include several phenomena known to be important for tumor development in other situations or over time scales longer than those considered in this study [10]. For example, because we considered only the first five days of tumor development, we did not include mechanisms for tumor mutation or phenotypic evolution, tumor chemotaxis, escalating invasion of neighboring tissue, or the adaptive immune response, because these phenomena are relevant over longer time scales. Furthermore, we do not consider other sources of heterogeneity within the TME, including the

ECM or surrounding epithelial tissue, which has previously been observed to interact strongly with heterogeneity in tumor cell phenotype [1], which we explicitly do not consider here. Variation in overall epithelium/ECM rigidity is captured indirectly by model parameter *p8*, which also captures how invasive the tumor is.

All parameter names (*p1-p18*), descriptions, and base parameter values are detailed in Table S1, which also includes citations to published literature and corresponding rationale for the base parameter values selected. Table S2 enumerates all core model assumptions along with corresponding rationale.

Macrophage polarization. Naïve macrophages become "activated" when exposed to factors such as bacterial endotoxin, or in the tumor environment, the endogenous protein high mobility group box 1 (HMGB1), which is released by necrotic cells and induces signaling via Toll-like Receptor 4 (TLR4) [11]. This "classically activated" state is termed M1. If activation is preceded by or concomitant with exposure to certain cytokines associated with immune suppression (most notably IL-10, but also IL-4 and IL-13), then macrophages polarize to an "alternatively activated" M2 state [6]. Such pro-M2 factors may be secreted by the tumor, by existing M2 cells, or by other immune cells at later stages of tumor development [6,12].

To capture this biology succinctly within the model, polarization was driven by two soluble factors – the "Activator signal" and the pro-M2 signal (M2S). The Activator signal was released by living and dying tumor cells (at the time of death) at rates *p6* and *p15,* respectively. The lumped effector cytokine M2S was secreted by tumor and M2 cells at rates set by *p3* and *p4*, respectively. Naïve macrophages became activated when the local concentration of Activator signal exceeded the threshold *p14*, and at that point polarization to an M1 or M2 state was also assigned. For simulations run under a regime of deterministic polarization, activated cells were assigned to M2 if the local M2S exceeded the threshold *p13*, and otherwise the cells polarized to an M1 state. For simulations in which polarization was assumed to be stochastic,

the probability of assigning a cell to M1 (*prob_M1*) or M2 (*prob_M2*) depended on local M2S, the threshold *p13*, and a polarization stochasticity parameter *p16* as follows:

$$prob\_M1 = 1 - \frac{1}{2}\left(erf\left(\frac{\left(\frac{MS2}{p13}\right) - 1}{p16} \right) + 1 \right)$$

$$prob\_M2 = \frac{1}{2}\left(erf\left(\frac{\left(\frac{MS2}{p13}\right) - 1}{p16} \right) + 1 \right),$$

where *erf* is the error function. Thus, each cell exposed to a concentration of activator above *p14* first attempted to polarize to the M1 state, and was successful with probability *prob_M1*. If this polarization attempt was unsuccessful, this cell subsequently attempted to polarize to the M2 state, and was successful with probability *prob_M2*. The probability of not polarizing to either state is the product of the two functions and has a max value of 0.25, although in the majority of cases considered a cell would polarize to one of these two states at the first possible time it could do so.

Tumor killing by M1 macrophages. The capacity of M1 cells to promote tumor cell killing was captured in the model via the lumped and diffusible factor, "tumor lethality signal" (TLS). TLS was secreted at a rate *p5* by M1 cells and, if tumor cells were exposed to local TLS levels above the threshold *p12*, the tumor cells died, releasing Activator signal as described above.

Macrophage chemotaxis. Macrophages chemotax along gradients of various chemokines, which are secreted by both tumors and M2 cells [6-8]. To represent macrophage chemotaxis succinctly, a combined chemokine function was lumped into M2S, since macrophage-attractant chemokines are secreted by M2 cells and tumors (both of which secreted M2S in the model). Macrophages are highly motile, and thus we assume that on the

time scales considered in our model, and in the presence of strong gradients of M2S (which are present in every simulation), macrophage net movement would be effectively deterministic along the direction of steepest local M2S gradient, at rate *p1*. As discussed in the main text, the addition of a stochastic component to macrophage chemotaxis did not substantially alter the results of our simulations (see Figure S3), which supports our use of a deterministic chemotaxis model.

Oxygen transport and consumption. Oxygen enters the TME through the vascular system, which becomes hyper-developed and disorganized at tumor sites via the action of secreted angiogenic factors such as VEGF [13-15]. In the model, vasculature was included as a non-diffusing environmental species (Figure 1C). Oxygen entered the TME at each lattice site at a rate proportional to the amount of vasculature. The vasculature, in turn, was deposited in proportion to the local level of lumped mediator M2S. All lattice sites (which initially represent background epithelial tissue) were assumed to include some vasculature at the time of simulation initialization, and given the relatively short time scale considered in our model, substantial vasculature remodeling was assumed not to occur. Thus, secreted VEGF (a factor that is represented by the lumped M2S signal) was assumed to enhance blood supply by modifying the existing vasculature without inducing substantial spatial remodeling. All cells within the TME consumed oxygen at the same rate (*p7*), and all cells died from anoxia when local oxygen levels reached zero.

Tumor growth. Given the short timescale of the questions investigated here (<5 days) and because we assumed that the surrounding epithelial cells and ECM were homogeneous in space surrounding the tumor, the simplest possible representation of tumor growth was used – phenotypically homogeneous, non-mutating cells dividing at a fixed rate (*p8*).

Macrophage recruitment. Only naïve macrophages were recruited to the TME (introduced to the simulation). Naïve macrophages entered via the vasculature (representing

recruitment of monocyte-derived macrophages, for example) in a stochastic fashion governed by *p9* and with a probability proportional to the vascularization at each lattice site.

Governing equations for secreted factor diffusion

$$\overbrace{\frac{\partial M}{\partial t}}^{\text{rate of change of M2S}} = \overbrace{p_2\nabla^2 M}^{\text{diffusion of M2S}} + \overbrace{p_3 T_{i,j}}^{\text{secretion by tumor cells}} + \overbrace{p_4 M2_{i,j}}^{\text{secretion by M2 cells}}$$

$$\overbrace{\frac{\partial A}{\partial t}}^{\text{rate of change of Activator}} = \overbrace{p_2\nabla^2 A}^{\text{diffusion of Activator}} + \overbrace{p_6 T_{i,j}}^{\text{secretion by tumor cells}} + \overbrace{p_{15} D_{i,j}}^{\text{secretion by newly dead tumor cells}}$$

$$\overbrace{\frac{\partial L}{\partial t}}^{\text{rate of change of TLS}} = \overbrace{p_2\nabla^2 L}^{\text{diffusion of TLS}} + \overbrace{p_6 M1_{i,j}}^{\text{secretion by M1 cells}}$$

$$\overbrace{\frac{\partial O}{\partial t}}^{\text{rate of change of Oxygen}} = \overbrace{(10\times p_2)\nabla^2 O}^{\text{diffusion of Oxygen}} + \overbrace{\frac{R}{p_7} V_{i,j}}^{\text{secretion by vasculature}} - \overbrace{R\times C_{i,j}}^{\text{Consumption of oxygen by all cells}}$$

In the above equations governing how the four different secreted factors diffuse, all parameters are referred to by their corresponding symbol (Table S1); the only exception is the parameter R, which governs the rate of oxygen consumptions and was kept fixed at R = 8 x 10^4 pg/(LS*s). *M* is the concentration of M2S, *A* is the concentration of Activator, *L* is the concentration of tumor lethality signal, and O_2 is the concentration of oxygen. $T_{i,j}$, $M2_{i,j}$, $M1_{i,j}$, and $D_{i,j}$ and $C_{i,j}$ represent the presence of individual tumor cells, M2 cells, M1 cells, newly dead tumor cells or any living cell type at lattice position *(i,j)*, respectively. $V_{i,j}$ is the value of the continuous, non-diffusing, vasculature field evaluated at *(i,j).* Given the base parameter values selected, diffusible molecules diffuse out of the TME within ~2 hours, which is shorter than the half-life of any of the molecules considered. Therefore, degradation of secreted factors was not included explicitly in these equations. The active uptake of oxygen by all cells is explicitly included in the model, because oxygen is known to be both a limiting resource within the TME and to be actively taken up by all cell types. However, active uptake of the three signaling molecules was not included,

since uptake of these molecules was assumed to have less of an effect on the overall intracellular concentrations of these species. We assumed that during the early time points of tumor growth simulated with our model, tumor-mediated impacts on immune function would be limited to the local environment (e.g., not systemic). Thus, in our model we assumed that at the boundary of our simulation region (and beyond), all tissue was healthy and not substantially affected by the presence of the tumor. Due to this, boundary conditions used for PDE calculations were fixed (Dirichlet type), and in particular, the value at the boundary for all four diffusible signals was fixed at the initial basal level of each individual signal (i.e., basal TLS and Activator concentrations were zero, the basal mean M2S concentration was set by the parameter *p11*, and the basal Oxygen concentration was set to be 30 pg/μL, which represented the steady state value of production vs. consumptions in the base set of parameter values.)

3. Simulation initiation and propagation

Each simulation run occurred on a two dimensional 100 x 100 square lattice (where each lattice site represents an area that is 10μm x 10μm), which represents a slice of epithelial tissue from a three dimensional TME [1]. This domain is substantially larger than the terminal size of any simulated tumor. In general, while 3-D models may add some realism to such a representation, they are substantially (>100x) more computationally expensive to run, and this cost would preclude most of the analyses performed here. Furthermore, 2-D lattice models have proven highly useful for generating experimentally verifiable predictions about tumor development [1-4]. Thus, a 2-D lattice was selected to match the modeling and analysis approach taken here.

Each initialization of the model began with a small, clonal tumor of cells (*p18* set the exact number of cells) in the center of a homogenous tissue pre-populated with naïve macrophages distributed randomly throughout the space with a uniform density (set by *p10*). Initially, there was neither Activator signal nor TLS present, and oxygen was homogeneously distributed throughout the system. M2S was distributed stochastically throughout the space according to a predefined concentration distribution with average value *p11* and standard deviation *p17*. Pre-existing M2S could represent either homeostatic levels of M2S or the impacts of systemic immune dysfunction, as might be expected when a tumor metastasizes to a new, distal site. This latter parameter (*p17*) altered the roughness of the initial gradients of M2S and thus effectively manipulated spatial heterogeneity in initial M2S. Because each of the three secreted soluble factors (M2S, TLS, Activator) represent proteins with comparable molecular weights, these factors were all assumed to diffuse at the same rate, *p2*. Oxygen, which is a substantially smaller molecule, was assumed to diffuse ten times faster, 10**p2* (see Table S1 for details). In this way a single parameter (*p2*) governs the rate of diffusion for all four secreted factors, and the relative rates of diffusion were held constant for all simulations.

Computational Resources. All simulations were performed in MATLAB 2013a (The MathWorks Inc.) on 2.83 GHz Intel Xeon processors. For each of the two ABM MPSA calculations, more than 213,000 runs of the model were performed, utilizing approximately 30,000 processor hours.

4. Algorithmic details

Also see Figure S2 for a schematic outline of this algorithm.

0. Initialization

- Set all parameters p1-p18 according to predefined values.
- Initialize cell type tumor with p18 cells in a centered, circular arrangement on the lattice.
- Seed cell type Naïve MP throughout the domain randomly with density p10.
- Initialize diffusible signal M2S throughout the domain at mean value p11 and variance p17 (the spatial heterogeneity parameter), drawn from a normal distribution.
- Initialize empty arrays of cell type M1 MP, dead cell, and M2 MP. Initialize uniform arrays of diffusible signals Activator, TLS, and Oxygen as well as the Vasculature field at basal values.

While time < t_{max}:

1. Diffusion of signals

- M2S, TLS and Activator diffuse according to the diffusion rate p2.
- Oxygen diffuses at rate 10 x p2.
- Diffusion is implemented through a forward-Euler method with fixed boundary conditions.

2. Angiogenesis, oxygen production, and oxygen absorption.

- The vasculature present at (i,j), $V_{i,j}$, increases proportional to the level of M2S at that lattice site.
- Oxygen is produced at each lattice site proportional to $V_{i,j}$.
- Any of cell types Naïve MP, M1 MP, M2 MP, or tumor present at lattice site (i,j) decrease local Oxygen levels at a rate proportional to p7.
- If any individual lattice site has Oxygen level below 0, all cells there convert to cell type dead cell.

3. Signal secretion

- M2 MP secrete M2S at rate p4.
- Tumor cells secrete M2S at rate p3.
- M1 MP secrete TLS at rate p5.
- Tumor cells secrete Activator at rate p6.
- Dead tumor cells secrete Activator at rate p15 (only in the time step immediately following death from hypoxia).

4. Tumor division

- Individual tumor cells divide according to the tumor division time p8, proportional to the number of available adjacent lattice sites.
- The vasculature level at any new tumor site is reduced to 0.

5. Macrophage chemotaxis

- All macrophage cell types (Naïve, M1 and M2 cells) chemotax deterministically along gradients of M2S at a rate determined by p1. Specifically, if in a given time step a cell is chosen to move, that cell will move to the adjacent lattice site from its current one with the largest value of M2S.

6. Tumor death from TLS

• If the level of TLS at a lattice site becomes larger than the tumor threshold for it (p12) and there is a tumor cell at that spot, that cell is converted to the dead cell type.

7. Macrophage polarization

• The set of Naïve MP at lattice site with a concentration of Activator higher than the macrophage activation threshold p14 are converted to the M1 MP cell type with probability

$$prob\_M1 = 1 - \frac{1}{2}\left(erf\left(\frac{\left(\frac{MS2}{p13}\right) - 1}{p16} \right) + 1 \right),$$

where *erf* is the error function. p13 controls the threshold for macrophage polarization while p16 controls the steepness of the probability function (i.e., the functional heterogeneity).

• The set of Naïve MP at lattice site with a concentration of Activator higher than the macrophage activation threshold p14 that did not polarize to the M1 MP cell type are converted to the M2 MP cell type with probability

$$prob\_M2 = \frac{1}{2}\left(erf\left(\frac{\left(\frac{MS2}{p13}\right) - 1}{p16} \right) + 1 \right)$$

8. Naïve macrophage recruitment

• Naïve MP are introduced to empty lattice sites at a rate p9, proportional to the level of vasculature at each individual lattice site.

time = time + time_step (set by p2).

END

5. References cited in Text S1

1. Anderson AR, Weaver AM, Cummings PT, Quaranta V (2006) Tumor morphology and phenotypic evolution driven by selective pressure from the microenvironment. Cell 127: 905-915.
2. Basanta D, Strand DW, Lukner RB, Franco OE, Cliffel DE, et al. (2009) The role of transforming growth factor-beta-mediated tumor-stroma interactions in prostate cancer progression: an integrative approach. Cancer Res 69: 7111-7120.
3. Scott JG, Hjelmeland AB, Chinnaiyan P, Anderson AR, Basanta D (2014) Microenvironmental variables must influence intrinsic phenotypic parameters of cancer stem cells to affect tumourigenicity. PLoS Comput Biol 10: e1003433.
4. Jiao Y, Torquato S (2011) Emergent behaviors from a cellular automaton model for invasive tumor growth in heterogeneous microenvironments. PLoS Comput Biol 7: e1002314.
5. Scott JG, Basanta D, Anderson ARA, Gerlee P (2013) A mathematical model of tumour self-seeding reveals secondary metastatic deposits as drivers of primary tumour growth. J R Soc Interface 10: 20130011.
6. Mantovani A, Sica A, Sozzani S, Allavena P, Vecchi A, et al. (2004) The chemokine system in diverse forms of macrophage activation and polarization. Trends Immunol 25: 677-686.
7. Biswas SK, Gangi L, Paul S, Schioppa T, Saccani A, et al. (2006) A distinct and unique transcriptional program expressed by tumor-associated macrophages (defective NF-kappaB and enhanced IRF-3/STAT1 activation). Blood 107: 2112-2122.
8. Allavena P, Sica A, Solinas G, Porta C, Mantovani A (2008) The inflammatory micro-environment in tumor progression: the role of tumor-associated macrophages. Crit Rev Oncol Hematol 66: 1-9.
9. Mantovani A, Sica A (2010) Macrophages, innate immunity and cancer: balance, tolerance, and diversity. Curr Opin Immunol 22: 231-237.

10. Deisboeck TS, Zhang L, Yoon J, Costa J (2009) In silico cancer modeling: is it ready for prime time? Nat Clin Pract Oncol 6: 34-42.

11. Campana L, Bosurgi L, Rovere-Querini P (2008) HMGB1: a two-headed signal regulating tumor progression and immunity. Curr Opin Immunol 20: 518-523.

12. Sato T, McCue P, Masuoka K, Salwen S, Lattime EC, et al. (1996) Interleukin 10 production by human melanoma. Clin Cancer Res 2: 1383-1390.

13. Roskoski R, Jr. (2007) Vascular endothelial growth factor (VEGF) signaling in tumor progression. Crit Rev Oncol Hematol 62: 179-213.

14. Owen JL, Mohamadzadeh M (2013) Macrophages and chemokines as mediators of angiogenesis. Front Physiol 4: 159.

15. Santin AD, Hermonat PL, Ravaggi A, Cannon MJ, Pecorelli S, et al. (1999) Secretion of vascular endothelial growth factor in ovarian cancer. Eur J Gynaecol Oncol 20: 177-181.
